# Encapsulating Textiles with Dynamic Covalent Networks for Sustainable and Efficient Oil Spill Cleanup

**DOI:** 10.1002/advs.202520228

**Published:** 2025-12-19

**Authors:** Changyi You, Ping Yu, Haiyue Wang, Qirui Huang, Wei Hong, Cai Liu, Qinchao Sun, Yan Wang, Youwei Ma, Zuming Hu

**Affiliations:** ^1^ School of Environmental and Chemical Engineering Jiangsu Ocean University Lianyungang China; ^2^ Institute of Materials École Polytechnique Fédérale de Lausanne (EPFL) Lausanne Switzerland; ^3^ State Key Laboratory for Modification of Advanced Fiber Materials, College of Materials Science and Engineering Donghua University Shanghai China; ^4^ Shandong Hualun Advanced Materials Co., Ltd. Linyi Shandong China

**Keywords:** a circular materials economy, chemically recyclable, dynamic covalent networks, oil spill remediation, textile composites

## Abstract

The development of high‐performance sorbents for oil spill remediation is highly desirable, which now calls for the integration of sustainable design in the context of advancing a circular materials economy. Here, we report a class of textile composites prepared through encapsulating industrially relevant fabrics with dynamic imine networks, which combine exceptional oil removal efficiency with intrinsic chemical recyclability. The dynamic imine networks are synthesized via a one‐pot polycondensation of terephthalaldehyde, isophorone diisocyanate, and a trifunctional amine cross‐linker. When coated onto fabrics such as PET, nylon, cotton, and polyimide, the resulting composites exhibit significant improvements in mechanical strength (8.6‐fold increase in stress at break), surface hydrophobicity (water contact angle of 123° compared to 0°), antifouling resistance, and oil sorption capacity (1.4–16‐fold increases in oil uptake). The high oil removal performance is retained across a broad temperature range (10–50 °C), and can be further improved by increasing the coating concentration, with the maximum sorption of 28.5‐fold the material's weight for silicone oil. Importantly, the coating is chemically recyclable, enabling efficient separation and recovery of both the fabrics and the dynamic networks without loss of material quality.

## Graphical abstract

By encapsulating industrially relevant fabrics with dynamic imine networks, we show that the resulting composites exhibit exceptional oil absorbtion capability and chemical recyclability. It is poised for sustainable oil spill cleanup.

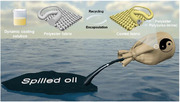

## 1 Introduction

Oil spills remain a serious global environmental threat, releasing millions of tons of petroleum into marine ecosystems annually as a result of offshore drilling accidents, tanker collisions, pipeline leaks, and natural disasters such as storms or earthquakes [1, 2]. According to the International Tanker Owners Pollution Federation Limited (ITOPF), over 5.8 million tons of crude oil have been spilled into the oceans from 1970 to 2022 [3]. It includes the gruesome Deepwater Horizon oil spill accident in 2010, leading to an estimated 280k tons of oil spill into the Gulf of Mexico [4]. These events spread rapidly over vast ocean regions, disrupting marine food chains, smothering coastal habitats, and introducing toxic hydrocarbons that can persist in the environment for decades [5, 6, 7]. Existing remediation techniques, including mechanical recovery (e.g., booms and skimmers), in situ burning, chemical dispersal, sorption, and membrane technology, show some effects, but they sometimes face limitations in efficiency, selectivity, environmental compatibility, or scalability, particularly under challenging sea and weather conditions [8, 9, 10, 11, 12, 13, 14]. Thus, developing oil spill cleanup techniques/materials that can simultaneously address these limitations is highly desirable and yet scientifically demanding.

Textile sorbents have emerged as promising materials for rapid and selective oil–water separation, since they integrate lightweight flexibility, high surface area, and engineered surface chemistry together [15, 16, 17]. Their excellent performance is usually rooted in coating with external hydrophobic polymers, as most textiles are intrinsically hydrophilic, exhibit limited oil uptake capacity, and degrade over prolonged exposure to saltwater, UV irradiation, and waves [18]. Indeed, polymer coatings can impart superhydrophobicity, oleophilicity, and enhanced durability to textile sorbents [19, 20]. The excellent durability typically necessitates the architecture of the coatings in cross‐linking networks, which show superior chemical stability and mechanical resilience than their linear counterparts [21]. However, the use of such cross‐linked architectures introduces end‐of‐life management challenges: their chemical inertness hampers the recyclability of their composites with textiles, resulting in substantial plastic waste and economic losses [22, 23].

Recent advancement in dynamic covalent networks (DCNs)—which are accessed by engineering dynamic covalent bonds (DCBs) to cross‐linked polymer networks through either *de‐novo* synthesis or post‐polymerization modification—provides an effective solution to the end‐of‐life recycling of thermosetting polymers [24, 25, 26]. The DCBs can undergo dissociative and/or associative exchange reactions, allowing the depolymerization and reprocessing of DCNs upon exposure to some stimuli (such as heat and light), which subsequently facilitate the recovery or reuse of their constituent components [27, 28, 29, 30, 31]. The DCBs reported so far include, but not limited to, imines [32, 33, 34, 35, 36, 37, 38], vinylogous urethanes [39, 40, 41, 42, 43, 44, 45], enamide [29, 46, 47, 48], dioxaborolanes [49, 50], and tri/diketoenamines [51, 52, 53, 54]. Among them, imine bonds (Schiff bases), first reported by Hugo Schiff in 1864, have garnered considerable attention in the synthesis of DCNs over the past two decades due to their good balance of chemical robustness and reversibility, coupled with high synthetic accessibility [32]. These attributes, in principle, unlock the potential of dynamic imine chemistry to access chemically robust and yet recyclable coatings for textile sorbents, even on an industrial scale, thereby contributing to a circular materials economy.

**SCHEME 1:**
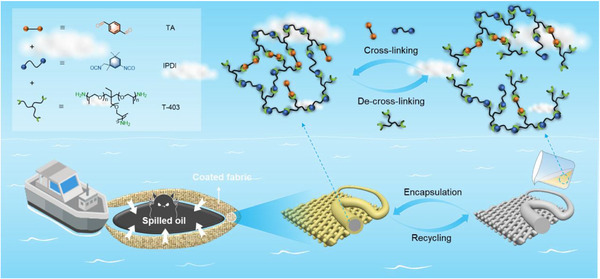
Illustration of the preparation of a textile composite by encapsulating a fabric with dynamic imine networks, synthesized from the polymerization of **TA**, **IPDI**, and **T‐403**, the application in marine oil spill cleanup, and the chemical recycling of the encapsulated fabric.

Despite the significant potential, to our knowledge, the marriage of dynamic imine chemistry and textile coating so far remains largely unexplored. To fill the gap, we here develop a new type of dynamic imine network‐coated textile composites that integrate high oil removal capability and chemical recyclability together. The dynamic imine networks are synthesized through one‐pot polycondensation of terephthalaldehyde (**TA**), isophorone diisocyanate (**IPDI**), and amine‐terminated trimethylolpropane tris[poly(propylene glycol)] (**T‐403**) (Scheme 1). The polymer networks produced are a class of cross‐linked poly(urea–imine)s (**CPUI**s), and they exhibit tunable thermal, mechanical, and thermomechanical properties through adjusting the feed ratios of the starting materials. Further coating them on industrially relevant polyethylene terephthalate (**PET**) fabrics through impregnation, the resulting composites show a core–shell structure, improved mechanical strength, and robustness as compared to the uncoated fabrics. Encapsulation also imparts significant enhancements in surface hydrophobicity and oil removal performance, with stabilized water contact angles reaching 130° (compared to 0° for uncoated **PET**) and 1.6–2.7‐fold increases in oil sorption capacity (OSC). The coated fabrics are excellent at removing industrial oils across a wide range of temperatures between 10 and 50 °C (Scheme 1), showing OSC values of 3.4–16.3 g g^−1^. This approach is versatile and readily extends to other fabrics, including nylon, cotton, and polyimide, enhancing their OSC by 40–1520%. Moreover, owing to the dynamic nature of the imine linkages, the textile sorbents are chemically recyclable through the exchange reaction with the amine functionality of **T‐403**, allowing the separation and reuse of both the fabrics and the **CPUI**s (Scheme 1).

## 2 Results and Discussion

### 2.1 Synthesis and Characterization of Cross‐Linked Poly(Urea–Imines)

**FIGURE 1:**
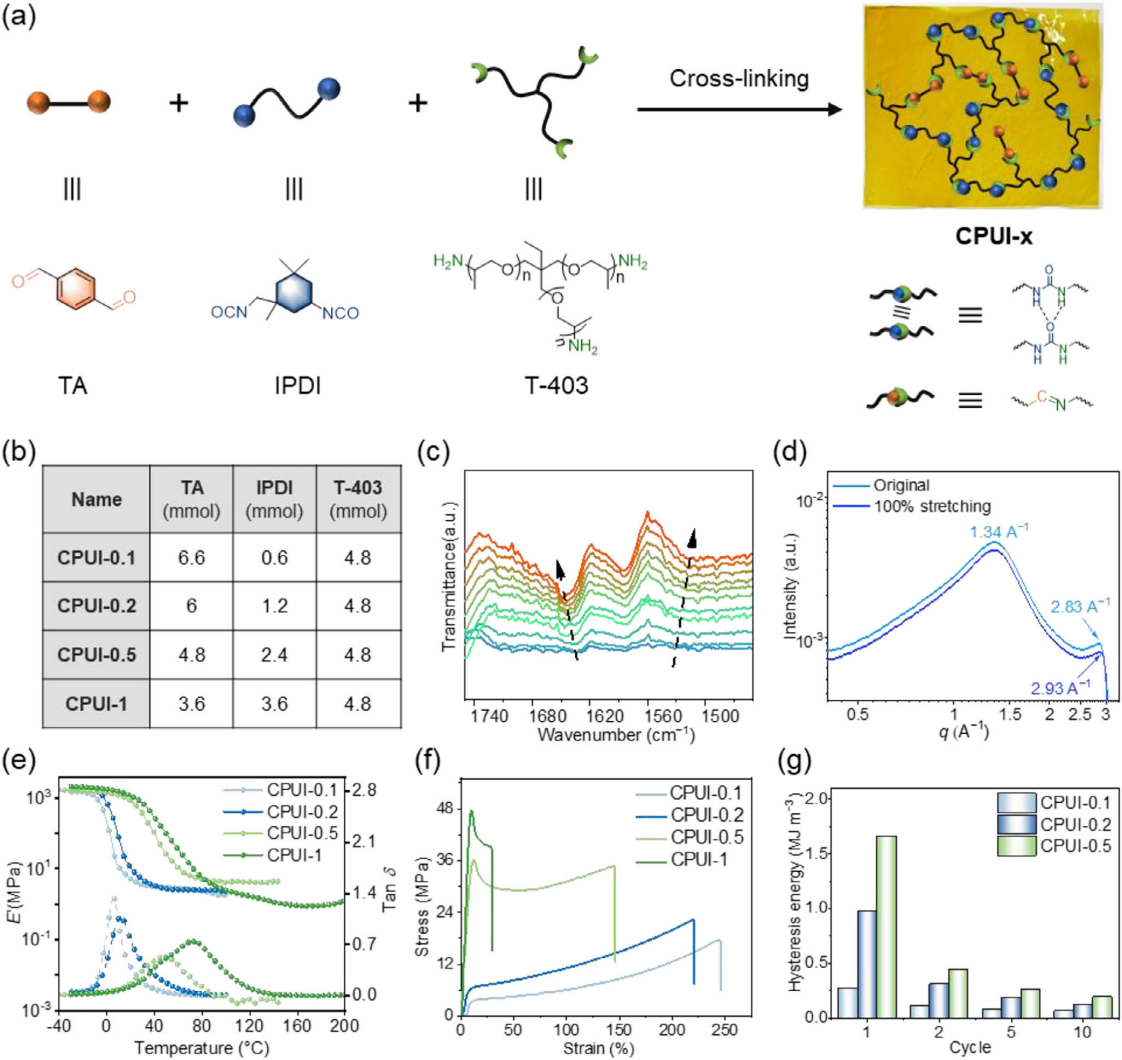
(a) Illustration of the synthesis of **CPUI‐x** networks by polymerization of **TA**, **IPDI**, and **T‐403**. (b) Recipes used in the synthesis of **CPUI‐x**. (c) Temperature‐variable FTIR spectra of **CPUI‐1** in the spectrum region between 1450–1750 cm^−1^. (d) WAXS profiles of the original **CPUI‐0.1** and the stretched **CPUI‐0.1** at 100% strain. (e) DMA traces and (f) stress–strain curves of **CPUI‐x** films. (g) Hysteresis energies of the first, second, fifth, and tenth cycles of **CPUI‐0.1/0.2/0.5** films during cyclic tensile testing at a maximum strain of 10%.

A series of **CPUI** networks (**CPUI‐x**) were synthesized by polymerizing **TA**, **IPDI**, and **T‐403** in one pot (Figure 1a), with the molar feed ratio (**x**) of **IPDI** to **TA** as 0.1, 0.2, 0.5 and 1, while keeping the amount of amine functionality equal to the sum of aldehyde and isocyanate groups (formulations shown in Figure 1b). The reactions lead to the formation of both imine and urea linkages, as evidenced by the emergence of vibration bands at 1634 and 1558 cm^−1^, ascribed to the stretching of ─C═N─ and ─C═O─, respectively, in the FTIR spectra of **CPUI‐0.1** (Figure S1a). The rationale of introducing urea motifs to the imine polymer networks arises from their propensity to form hydrogen bonds and resonance‐induced high stability [25, 47], which potentially contribute to the improvement in both performance and stability of **CPUI‐x**. Upon increasing the **IPDI** content, more urea bonds form (Figure S1b).

The presence of hydrogen bonding in **CPUI‐x** was examined by subjecting **CPUI‐1** to temperature‐variable FTIR from 40 to 150 °C. The vibration peaks at 1634 and 1530 cm^‒1^ ascribed to the ─C═O─ stretching and N─H bending of urea, undergo blue and red shifts, respectively (Figure 1c), which strongly support that hydrogen bonds exist in the networks and are gradually dissociated at elevated temperatures. Moreover, Atomic Force Microscopy (AFM) analysis of the samples reveals the formation of phase separation in **CPUI‐0.1** and **CPUI‐1**, with the latter displaying more uniformly distributed domains (Figures S2 and S3). Further observation of **CPUI‐0.1** using Wide‐Angle X‐ray Scattering (WAXS) shows that it exhibits two sharp Bragg peaks at q* = 1.34 and 2.83 A^‒1^ (Figure 1d), corresponding to periodicities (*D*) of ca. 0.47 and 0.22 nm (calculated as *D* = 2π/q*), respectively. Their formations are probably induced by π–π stacking interactions of **TA** units and hydrogen bonding among urea moieties [55, 56].

The thermal properties of **CPUI‐x** were then evaluated by Thermogravimetric Analyses (TGA) and Differential Scanning Calorimetry (DSC). TGA curves reveal that all samples exhibit excellent thermal stability, with 5% decomposition temperatures (*T*_5%_) of 325–335 °C (Figure S4). The difference lies in the residual char ratio above 600 °C; As the value **x** increases from 0.1 to 1, the char ratio of **CPUI‐x** gradually decreases from 25% to 8%, which we attribute to the reduced aryl ring content in the networks. Meanwhile, their glass transition temperature (*T*_g_) increases from 21 °C of **CPUI‐0.1** first to 29 °C of **CPUI‐0.2**, then to 44 and 66 °C of **CPUI‐0.5** and **CPUI‐1**, respectively (Figure S5). The enhanced *T*_g_ probably results from the more restricted chain mobility in the networks featuring a larger amount of urea bonds, which form more hydrogen bonds.

We next investigated the thermomechanical properties of **CPUI‐x** films by Dynamic Mechanical Analysis (DMA). All films exhibit a discernible decline in storage modulus (*E*’) between 6 and 73 °C, in agreement with the appearance of a peak in the tan δ plots, which is associated with the polymers’ *T*_g_ (Figure 1e). Both the onset of *E*’ decline and the tan δ peak shift to a higher temperature with increasing urea content (i.e., higher **x** values). For example, it is 6 °C for **CPUI‐0.1** and slightly increases to 13 °C for **CPUI‐0.2**, prior to reaching 41 and 73 °C of **CPUI‐0.5** and **CPUI‐1**, respectively (Figure 1e). This result demonstrates an increase in *T*_g_, aligning well with the trend found in the DSC analysis (Figure S5). Following the *T*_g_‐induced decline in *E*’, a rubbery regime appears in all **CPUI‐x** films, indicative of their cross‐linked network structure. Within the regime, the *E*’ values converge to approximately 2.5 MPa across all samples, suggesting comparable cross‐link densities *ν*_e_ regardless of composition (Figure 1e). Following the theory of rubber elasticity [42], calculation on *ν*_e_ shows that they are 3.7×10^2^, 3.1×10^2^, 4.1×10^2^, and 3.1×10^2^ mol m^‒3^ for the **CPUI‐x** films with **x** = 0.1, 0.2, 0.5, and 1 in sequence.

The mechanical properties of **CPUI‐x** were first studied by uniaxial tensile testing (Figure 1f). Distinct differences in tensile behavior are observed between low‐ and high‐**IPDI**‐content samples; **CPUI‐0.1/0.2** samples present non‐linear stress–strain curves with strains at break of 220–246%, characteristic of elastomeric behavior, while **CPUI‐0.5/1** films display plastic‐like tensile behavior, featuring an initial elastic regime, yielding at 10% strain, followed by plastic deformation (Figure 1f). Further analysis shows that increasing the value **x** results in increased stress at break but compromised strain at break; Upon increasing **x** from 0.1 to 1, the stress at break first sees a slight increase from 17 MPa of **CPUI‐0.1** to 22 MPa of **CPUI‐0.2**, then significantly rises to 34 MPa of **CPUI‐0.5** and finally to 39 MPa of **CPUI‐1** (Figure 1f). Concurrently, the strain at break decreases from 246% of **CPUI‐0.1** to 29% of **CPUI‐1** (Figure 1f). These trends are attributed to enhanced hydrogen‐bonding interactions, which strengthen the networks at the expense of chain mobility. Two additional networks featuring only imine or urea motifs were synthesized as the control samples (Figure S6a–c). Among them, the pure polyimine networks are weaker than **CPUI‐0.1** in terms of both stress and strain at break (Figure S6d), while the pure polyurea networks are too brittle to be measurable by tensile testing. Such comparisons indicate that the joint presence of imine or urea linkages contributes to the good mechanical robustness of the **CPUI‐x** networks. This is further corroborated by the result that well balance of composition of these two chemical motifs leads to a high toughness of 44 MJ m^‒3^ seen in the **CPUI‐0.5** network (Figure S7). Moreover, WAXS was used to observe the stretched **CPUI‐0.1** at 100% strain and shows that the scattering peak at q* = 1.34 A^‒1^, probably resulting from hydrogen‐bonded urea packing [57, 58], remains unchanged whereas the domain at q* = 2.83 A^‒1^ slightly shifts to 2.93 A^‒1^ (Figure 1d), indicating a minor decrease in *D* from 0.22 to 0.21 nm under tensile loading.

We then subjected **CPUI‐x** to cyclic tensile testing (Figure S8), in which the samples were repeatedly stretched and released to a maximum strain of 10% for 10 cycles. All films exhibit a pronounced hysteresis loop during the first cycle, followed by progressive narrowing in subsequent cycles (Figure S8). Integration of the enclosed area of the hysteresis loop allowed us to calculate the hysteresis energy (*Δ*W) of **CPUI‐x** for each cycle, which quantifies the energy dissipated per cycle (Figure 1g). The results demonstrate that *Δ*W in the first cycle is the largest for each **CPUI‐x** sample, ranging between 0.3 and 1.7 MJ m^‒3^, and increases with higher **x** values, suggesting greater energy dissipation probably due to increased hydrogen‐bond interactions. In the second cycle, *Δ*W decreases substantially relative to the first one, from 0.3, 1.0, and 1.7 MJ m^−3^ for **CPUI‐0.1**, **CPUI‐0.2**, and **CPUI‐0.5**, respectively, to 0.1, 0.3, and 0.4 MJ m^−3^. Further cycling to the fifth and tenth repetitions results in only minor reductions in *Δ*W (Figure 1g), indicating stabilization of the network's energy dissipation behavior.

### 2.2 Preparation and Characterization of CPUI‐0.1‐Coated PET Fabrics

**FIGURE 2:**
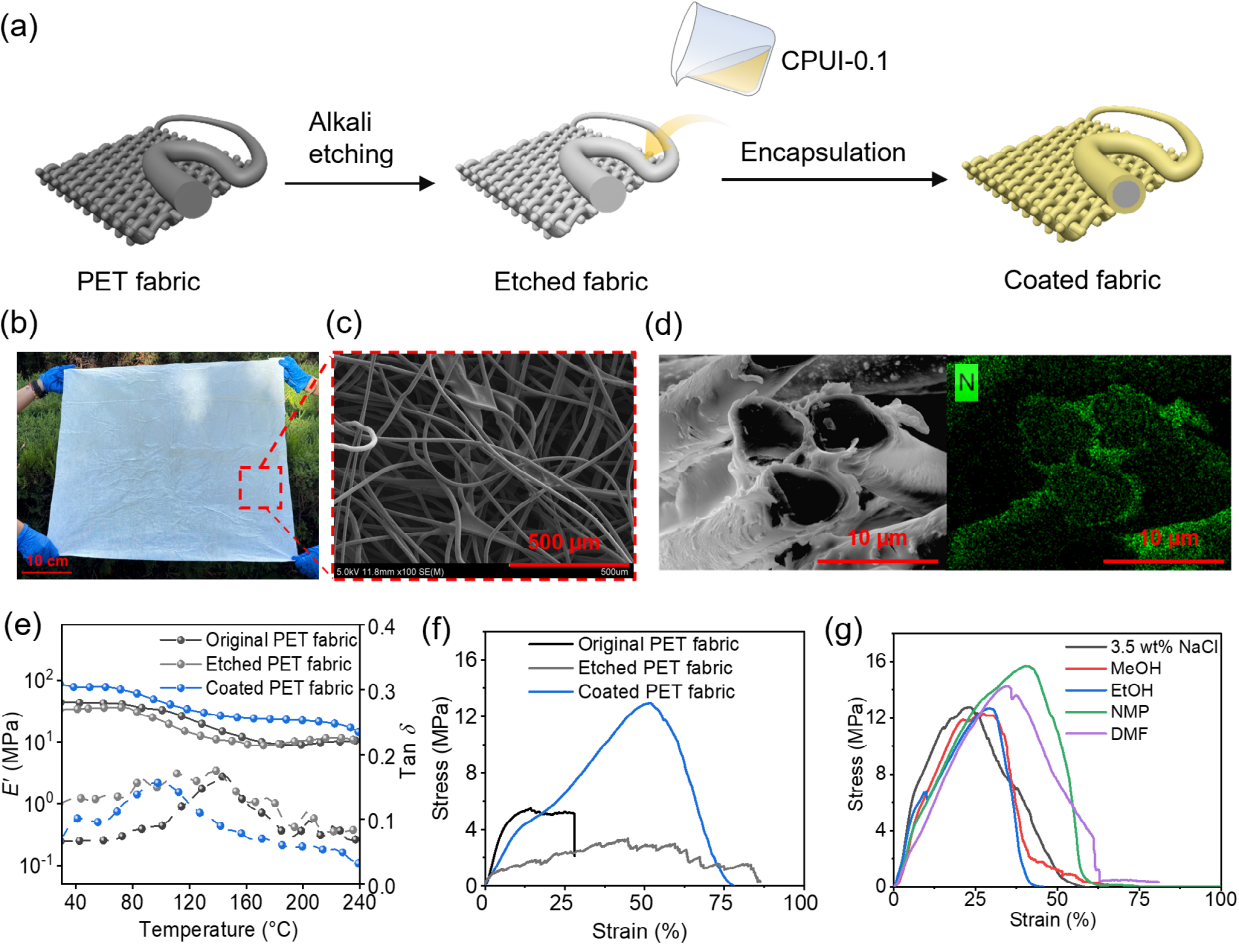
(a) Illustration of the preparation of a **CPUI‐0.1**‐coated **PET** fabric through first etching **PET** fabric using alkali, followed by encapsulation of the etched fabric with **CPUI‐0.1** solution. (b) Photograph and (c) SEM image of a coated **PET** fabric. (d) SEM (left) and EDX (right) images of the cross‐section of the coated **PET** fibers. (e) DMA traces and (f) stress–strain curves of the original, alkali‐etched, and coated **PET** fabrics. (g) Stress–strain curves of the coated **PET** fabrics after immersion in salty water or various solvents for 24 h.

After successfully synthesizing the **CPUI‐x** networks, we next investigated their application in the preparation of textile composites through a straightforward impregnation approach. **PET** fabrics with diameters of 13–21 µm were selected as the representative. As shown in Figure 2a, they were first etched by sodium hydroxide (1.25 wt.%) at 75 °C for 2 h before soaking in 1 wt.% **CPUI‐0.1** precursor solution for 12 h and subsequent drying at 80 °C overnight (Figure 2a). The initial alkali‐etching treatment is to create more carboxylate and hydroxyl groups on the fiber surface—a treatment that is widely adopted to prepare fiber composites—, and that possibly enhances the interfacial adhesion with the coating through hydrogen‐bonding interactions [59, 60, 61]. The choice of **CPUI‐0.1** as the coating substrate is rooted in its high hydrophobicity, as reflected by its largest water contact angles as compared to other **CPUI‐x** networks (Figure S9). The process is scalable and can produce **CPUI‐0.1**‐coated **PET** fabrics with a large size, good flexibility, and light weight (Figure 2b; Figures S10 and S11). Scanning Electron Microscopy (SEM) and Polarized Optical Microscopy (POM) analyses reveal that the porous network structure of the **PET** fabrics is well‐preserved after coating (Figure 2c), but the fiber surfaces turn from smooth to rough (Figures S12 and S13), suggesting non‐uniform deposition of **CPUI‐0.1**. The fiber composite exhibits a core–shell structure with the shell thickness of 1.2–1.9 µm (Figure S14), as confirmed by SEM and Energy Dispersive X‐ray Spectroscopy (EDX) (Figure 2d).

The thermal properties of **CPUI‐0.1**‐coated **PET** fabrics were studied by TGA analysis and compared with those of etched fabrics (Figure S15). The two fabrics exhibit a similar *T*_5%_ of ca. 420 °C, but differ in the final char ratio above 700 °C, with the higher value (25% *vs* 13%) seen in the coated fabric as compared to the etched one (Figure S15). DMA was then used to evaluate the thermomechanical properties of the fabrics before and after the alkali etching and encapsulation treatments (Figure 2e). The original **PET** fabric exhibits a *E*’ decline and a tan δ peak at 144 °C, which is shifted to a lower temperature of 114 °C after the etching treatment (Figure 2e), indicative of decreased *T*_m_ due to partial hydrolysis of **PET**. Upon encapsulation, although the *T*_m_ still sees a decrease to 98 °C, the *E*’ is significantly enhanced across the measured temperature range; For example, they are 44 and 34 MPa at 40 °C for the original and etched **PET** fabrics, respectively, and increase to 77 MPa of the coated one (Figure 2e). This highlights the critical role of the **CPUI‐0.1** coating in improving the thermomechanical performance of **PET** fabrics.

The mechanical properties of the original, etched, and coated **PET** fabrics were analyzed by tensile testing (Figure 2f). The stress–strain curves show that the original **PET** fabric exhibits a low stress at break of 5.1 MPa, which becomes even weaker and decreases to 1.5 MPa following alkali etching (Figure 2f). In contrast, encapsulation with 1 wt.% **CPUI‐0.1** solution leads to a significant increase in stress at break to 12.9 MPa, representing 2.5‐ and 8.6‐fold improvements relative to the original and etched fabrics, respectively (Figure 2f). During tensile deformation, we found that the fibers at the edges of the original and etched **PET** fabrics are torn apart first, generating cracks that propagate transversely into the interior (Figure S16a,b). By comparison, the coated fabric maintains structural integrity prior to its breakage (Figure S16c). These observations indicate that the **CPUI‐0.1** coating serves as a binder, integrating discrete fibers into a cohesive network, thereby preventing premature cracking and substantially enhancing mechanical strength. Impressively, such high strength enabled the coated fabric of a size of 9.57 cm × 2.4 cm × 0.15 cm (*l* × *w* × *h*) to hold a 15 kg water bottle and even a 54 kg person without failure (Figure S17).

The mechanical resilience of the coated fabrics was tested by first immersing them in saline water and various organic solvents, including methanol, ethanol, NMP, and DMF for 24 h, followed by drying at 70 °C to remove the solvents. Tensile testing was performed after each treatment, and the resulting stress–strain curves were compared with those of the untreated coated fabric (Figure 2g; Figure S18). The results demonstrate that exposure to these solvents, either with or without drying, has minimal impact on the mechanical performance, as evidenced by the comparable stress and strain at break (Figure 2g; Figure S18), reflecting the excellent mechanical robustness of the materials. It further indicates that our coating strategy is effective in ensuring good environmental resistance to the textiles, which is one of the foundational pillars toward their application in complex and harsh marine conditions.

### 2.3 Hydrophobicity, Antifouling, and Oil Removal Capabilities of Coated PET Fabrics

**FIGURE 3:**
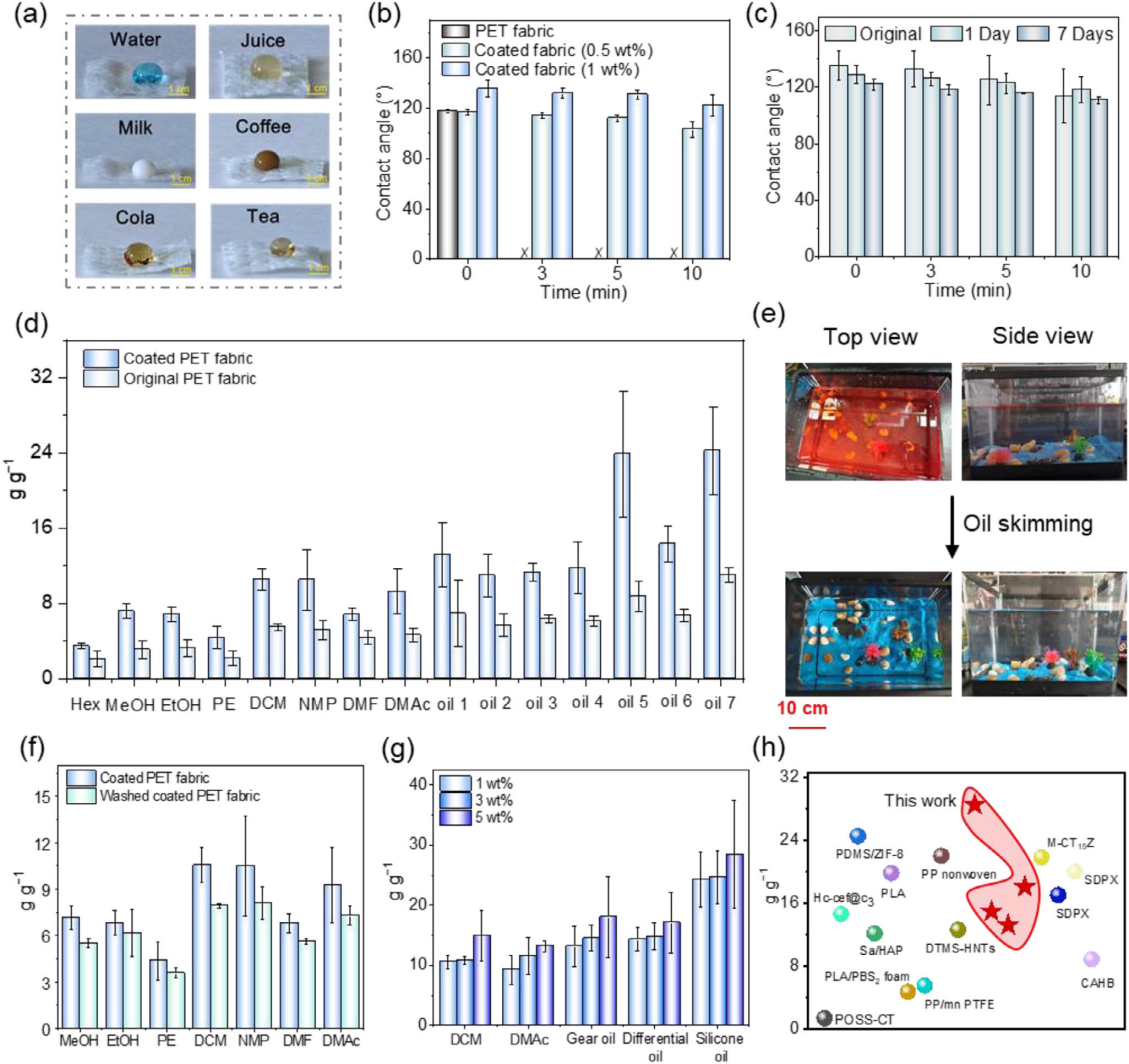
(a) Photographs of various aqueous droplets standing on coated **PET** fabrics. (b) Variation of contact angles over time of the original and **CPUI‐0.1**‐coated **PET** fabrics. (c) Variation of contact angles over time of the original, and the 1 wt.% **CPUI‐0.1**‐coated **PET** fabrics after the exposure to 85 %RH at 25 °C for 1 or 7 days (as indicated). (d) Histogram of the OSC of the original and **CPUI‐0.1** (1wt.%)‐coated **PET** fabrics toward organic solvents and industrial oils (as indicated), with the oil 1 to 7 corresponding to gear oil, hydraulic oil, brake fluid, automatic transmission fluid, lubricant, differential oil, and silicone oil in sequence. (e) Photographs showing the changes of hexane‐polluted water in a tank before (top) and after (bottom) treatment with a coated **PET** fabric, with the top and side views shown on the left and right sides, respectively (as indicated). Hexane is stained by Sudan red for illustration. (f) Histogram of the OSC of the **CPUI‐0.1** (1wt.%)‐coated **PET** fabric before and after being treated by simulated waving for 6 h. (g) Histogram of the OSC of the **CPUI‐0.1**‐coated **PET** fabrics, with the coating concentration of 1, 3, or 5 wt.%. (h) Comparison in OSC toward DCM, DMAc, differential, and silicone oils among the **CPUI‐0.1** (5wt.%)‐coated **PET** fabrics and other recently reported sorbents.

We next explored the potential of the coated fabrics as sorbents for marine oil removal, which in essence requires the materials to be superhydrophobic and oleophilic. The hydrophobicity was initially assessed by placing droplets of various aqueous solutions—including dyed water, juice, milk, coffee, cola, and tea—onto the fabric surfaces (Figure 3a). In all cases, the droplets display a near‐spherical shape without spreading, indicating excellent water repellency (Figure 3a). Further analysis using contact angle measurements shows that 0.5 wt.% **CPUI‐0.1**‐coated fabric exhibits an initial contact angle (0 min) of 117°, comparable to that of neat **PET** fabric (Figure 3b). However, prolonging the contacting time to 3 min leads to the disappearing of the droplet on the neat fabric, yet only a slight decrease of the contact angle to 114° for the coated material. The contact angle still reaches a value of 104° after an additional 7 min contact, demonstrating sustained non‐wetting behavior (Figure 3b). Increasing the coating concentration to 1 wt.% further enhances hydrophobicity, with a contact angle of 136° at 0 min, and a retained value of 123° after 10 min (Figure 3b). Intriguingly, measured at the same time, the contact angles of the coated fabrics, regardless of the coating concentration, both exceed that of a **CPUI‐0.1** film (Figure S19). We attribute the higher values to the presence of the mesh‐like architecture and roughened fiber surfaces in the coated fabrics (Figures S12 and S13).

The impact of moisture on the hydrophobicity of the coated fabrics was explored by exposing a 1 wt% **CPUI‐0.1**‐coated fabric to a high‐humidity environment (85 %RH) for 1 and 7 days, followed by contact angle measurements. As shown in Figure 3c, the initial contact angle (0 min) decreases slightly from 136° to 129° and 122° after 1 and 7 days, respectively. However, after 10 min contact time, the three samples exhibit similar contact angles, all around 115° (Figure 3c), demonstrating the retainment of the excellent hydrophobicity, and that high moisture has a negligible impact on the surface properties of the coated fabrics. The superhydrophobicity further imparts notable antifouling resistance to the materials, which is evidenced by several qualitative experiments. These include when we either poured stained water onto a coated fabric or immersed it inside coffee, neither of them caused significant contamination to the fabrics (Figure S20 and Videos S1 and S2). In stark contrast, identical treatments on uncoated **PET** fabrics result in obvious staining and contamination (Figure S20 and Videos S1 and S2), highlighting the protective effect of the **CPUI‐0.1** coating.

The oil removal capability of the fabrics was determined by measuring the weight of a 2 cm × 2 cm (*l* × *w*) fabric before and after its immersion in various organic solvents and industrial oils for 2 min. The oil sorption capability (OSC) was then calculated by the equation: OSC = (*W*_f_ −*W*_i_)/*W*_i_, where *W*_i_ and *W*_f_ represent the initial and final weights of the fabric, respectively. As shown in Figure 3d, neat **PET** fabrics exhibit OSC values of 2–11 g g^−1^, with the lowest and highest sorption observed for hexane and silicone oil (oil 7), respectively. Upon soaking in 1 wt.% of **CPUI‐0.1** solution, the coated fabrics see improvements in OSC toward all tested liquids, reaching 1.6–2.7 folds those of the uncoated fabrics. Among them, the highest improvement is seen in the removal of lubricants (oil 5), where the coated fabric absorbs 23.9 times its own weight, compared to only 8.7 times for the uncoated **PET** (Figure 3d). Moreover, the coated fabric far outperforms the neat **CPUI‐0.1** film in removing the same oils, with the latter only showing OSC values of 0.1–3.4 g g^−1^ (Figure S21), suggesting the significance of fabric texture in oil removal. Further comparisons with the neat polyimine‐ and polyurea‐coated **PET** fabrics at the coating concentration of 1 wt.% reveal that they exhibit comparable OSC values in removing DCM and DMAc, while **CPUI‐0.1**‐coated sample exceeds in absorbing industrial oils (Figure S22).

The coated fabrics show effectiveness in removing both heavy and light oils, no matter whether the oils float on or sink below water. This is reflected in two experiments when we used coated fabrics to approach DCM and hexane in their mixture with water, both solvents were absorbed into the fabrics within 5 s, with the materials residing in the same layer to the solvents (Videos S3 and S4). However, without the **CPUI‐0.1** coating, the fabrics fail to remove either solvent under identical conditions (Videos S3 and S4). These preliminary results encouraged us to address more practical issues. Specifically, 90 mL of dyed hexane was combined with 7 L of water in a fish tank with a dimension of 31 cm × 18 cm × 20 cm (*l* × *w* × *h*) (Figure 3e, top). A 35 cm × 35 cm (*l* × *w*) coated fabric was then used to skim the hexane. After three passes, totaling 35 s of treatment, the tank was effectively cleaned (Figure 3e, bottom; Video S5). To further optimize the oil sorption process, we then utilized the coated fabric as a filler material for continuous oil separation from water with the assistance of a vacuum pump operating at 0.05 MPa. Using this technique, 4.3 g of coated **PET** fabric is able to separate 700 mL of hexane from 7 L of water within 70 s (Video S6). In contrast, the same weight of unmodified **PET** fabric reaches its capacity when extracting only 100 mL of hexane under identical pumping conditions (Video S7). These findings support the practical applicability of the coated fabrics for rapid oil removal in realistic scenarios.

To assess the potential impact of waving, commonly seen in the oceans, on the coated fabrics, we first simulated waves by dropping water onto the fabric at a flow rate of 1.5 L min^−1^ from a height of ca. 25 cm for 6 h (Figure S23) [62]. The OSC of the treated fabric was further examined and compared with that of the untreated sample (Figure 3f); It shows that such treatment indeed reduces the oil removal capability, but only moderately, by less than 20%. Temperature also fluctuates from time to time in marine environments. Its impact was evaluated by measuring the OSC of the coated fabrics at different temperatures, including 10 °C, 25 °C, and 50 °C (Figure S24). Upon increasing the temperature, the OSC of the coated fabrics toward all oils tested first sees a decrease followed by an increase. For example, the OSC toward NMP is 16 g g^−1^ at 10 °C, decreasing to 10 g g^−1^ at 25 °C, then increasing to 13 g g^−1^ at 50 °C. The relatively high performances achieved at 10 and 50 °C are probably attributed to the reduced oil viscosity at the low temperature [63] and the enhanced polymer chain mobility at the high temperature [64], respectively.

The above results strongly support that coating with **CPUI‐0.1** helps **PET** fabrics to remove oils more efficiently. We next sought to further optimize the performance by increasing the coating concentration. Indeed, soaking in higher concentrations of **CPUI‐0.1** allows to increase OSC toward various oils (Figure 3g); The OSC values are 10–24 g g^−1^ for the fabric soaked in 1 wt.% **CPUI‐0.1**, and slightly rise to 11–25 g g^−1^ upon increasing the concentration to 3 wt.%, which further increase to 15–29 g g^−1^ when the concentration is 5 wt.%. This corresponds to the absorption of up to 29 folds the material's own weight toward oil. Encouraged by these results, we benchmarked the 5 wt.% **CPUI‐0.1**‐coated **PET** fabrics against other polymeric sorbents recently reported in the literature, including (coated) textiles [65, 66, 67, 68, 69, 70], particles [71, 72], and foams [73, 74, 75, 76, 77, 78, 79]. The comparison (Figure 3h; Table S2) reveals that our coated fabrics can rival and even surpass most state‐of‐the‐art sorbents in OSC, underscoring their superior oil uptake capability and strong potential for practical deployment.

The versatility of the coating strategy in the synthesis of high‐performance textile sorbents was explored by extending its application to nylon, polyimide, and cotton fabrics. Clearly, after the encapsulation with **CPUI‐0.1** at the 1 wt.% concentration, all three coated fabrics see improvements in OSC, increasing by 40%–1520% in comparison to their uncoated counterparts (Figures S25–S27). Particularly, the neat nylon fabric exhibits negligible affinity for DCM, with an OSC of only 0.22 g g^−1^, and after coating with **CPUI‐0.1**, which significantly increases to 3.67 g g^−1^—signifying an over 16‐fold improvement (Figure S25). These findings reflect that our **CPUI** coatings are effective in improving the oil removal performance of diverse textile substrates.

### 2.4 Recycling of CPUIs and their Coated PET Fabrics

**FIGURE 4:**
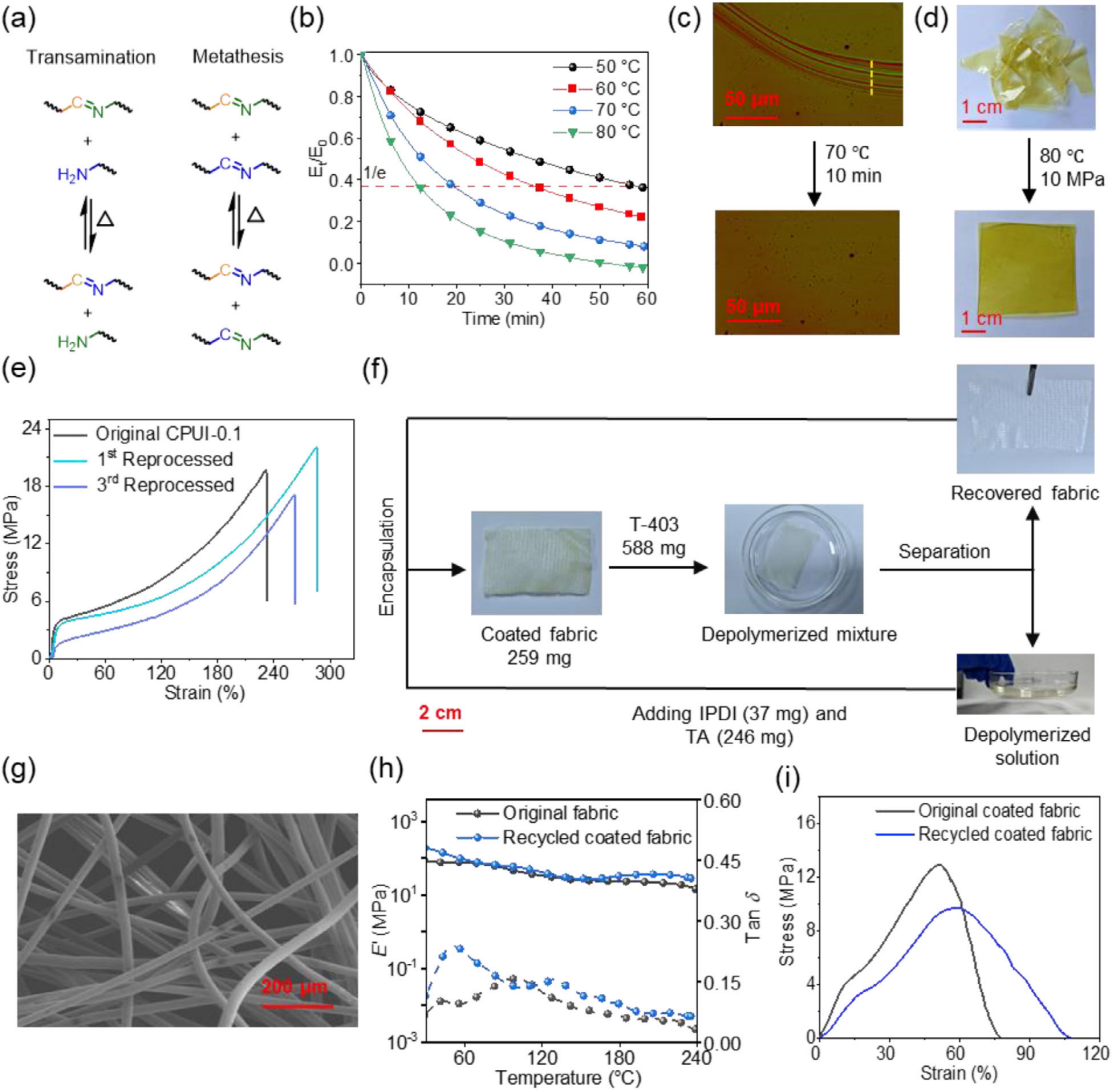
(a) Transamination (left) and metathesis (right) reactions involving the exchange of an imine with an amine or another imine, respectively. (b) Stress relaxation curves of **CPUI‐0.1** as a function of time. (c) Optical microscopy images of a scratched **CPUI‐0.5** film being healed at 70 °C for 10 min. (d) Photographs showing the reprocessing of **CPUI‐0.1** from small pieces into a new film by compression molding. (e) Stress–strain curves of the original, the first, and the third reprocessed **CPUI‐0.1** films. (f) Photographs showing chemical recycling of a coated **PET** fabric, involving depolymerization with **T‐403**, separation of the fabric and the depolymerized solution, followed by encapsulation of the recovered fabric with the repolymerized polymer solution. (g) SEM image of the recovered **PET** fabric. (h) DMA traces and (i) stress–strain curves of the original and resynthesized coated **PET** fabrics.

Having confirmed the excellent performance, including strong mechanical resilience, superhydrophobicity, and excellent OSC of our textile sorbents, we next explored their recyclability, which is rooted in the dynamic nature of imine bonds. The imine bonds can undergo transamination with free amines and bond metathesis between themselves (Figure 4a) [29, 37, 38]. These dynamic exchange processes enable **CPUI**s to reorganize their network structures under thermal conditions—a property not attainable in conventional thermosetting polymers [26]. Such a feature can be further translated to thermally repairing, reprocessing, and depolymerizing **CPUI**s for either prolonging their service life or enabling efficient material recovery and reuse.

The network rearranging ability of **CPUI‐x** was initially assessed by stress relaxation experiments conducted at 50 °C–80 °C (Figure 4b; Figure S28a). Both **CPUI‐0.1** and **CPUI‐0.2** exhibit efficient stress dissipation under thermal treatment, with faster relaxation observed at higher temperatures. (Figure 4b; Figure S28a). For example, the relaxation time (τ), at which the stress is dissipated to 1/e of its initial value, is 56 min at 50 °C for **CPUI‐0.1**, significantly decreasing to 19 min at 70 °C. Further increasing the temperature to 80 °C leads to the decrease in τ to only 12 min (Figure 4b). Arrhenius plots of ln(τ) against 1/*T* allowed us to calculate the activation energy (*E*_a_) for the stress relaxation (Figure S28b). **CPUI‐0.1** and **CPUI‐0.2** show close *E*_a_ values of 50 and 57 kJ mol^‒1^, respectively. We attribute the slightly lower *E*_a_ in **CPUI‐0.1** to its larger content of imine linkages (Figure 1b), which promotes more rapid network rearrangement and flowability.

The thermal repairability of **CPUI‐x** was evaluated by first scratching a **CPUI‐0.5** film in a width of ca. 20 µm. The scratched film was subsequently heated at 70 °C, and the scratch disappears after 10 min, as observed by optical microscopy (Figure 4c), indicating the good repairability upon thermal treatment. We then explored the weldability of **CPUI‐x** networks by fabricating a plastic bag from two rectangular **CPUI‐0.1** films with a size of 8 cm × 5 cm (*l* × *w*). A smaller **PET** sheet was placed between the films, with their edges overlapped along three sides (∼50 mm width). The assembly was clamped and heated at 100 °C for 1 h, yielding a welded bag with the final dimension of 7.5 cm × 4.5 cm (*l* × *w*). The bag is capable of holding both solid and liquid contents without leakage (Figure S29). These two properties provide the opportunity to maintain the structural integrity of **CPUI‐x** materials after their accidental damage, thereby extending their functional lifetime.

Besides these, the recyclability of **CPUI‐x** was also investigated through thermal reprocessing and amine‐induced depolymerization. As shown in Figure 4d, small pieces of **CPUI‐0.1** were compression‐molded at 80 °C under 10 MPa for 10 min, which successfully furnished a new film. The processes were repeated three times, and the first‐ and third‐time reprocessed films were subjected to tensile testing, with the results compared with that of the original material (Figure 4e). After three reprocessing cycles, the film retains excellent mechanical performance, with stress and strain at break values of 17 MPa and 260%, respectively, confirming their high reprocessability. The second recycling approach involves first depolymerizing small pieces of **CPUI‐0.1** (359 mg) through the reaction with newly added **T‐403** (1408 mg) at 60 °C, followed by the repolymerization with additional **IPDI** (89 mg) and **TA** (590 mg) (Figure S30). The processes regenerated a **CPUI‐0.1** film, tensile testing analysis of the film shows good mechanical recovery in comparison to the original one (Figure S31).

Among these strategies, the depolymerization/repolymerization protocols are highly relevant in recycling composites, since the depolymerization can transform cross‐linked networks into either linear or branch derivatives, which unlocks the potential to separate the polymer matrixes from the composite fillers through selective solvent dissolution [26, 28, 38, 80, 81]. To verify this, in the final part of our work, we endeavored to recycle the coated **PET** fabric following similar procedures to the recycling of **CPUI‐0.1**. Specifically, 588 mg of **T‐403** was added to 259 mg of coated **PET** fabric and heated at 60 °C to depolymerize the coating (Figure 4f). After 1 h, the fabric color changed from light yellow to off‐white, and SEM analysis reveals a smooth fiber surface (Figure 4g), both suggesting the removal of the coating. The fabric was then taken out and socked in the liquid mixture, obtained by the addition of 37 mg of **IPDI** and 246 mg of **TA** to the depolymerized solution (Figure 4f). Subsequent curing the soaked fabric at 70 °C produced a new coated **PET** fabric with the rough fiber surface (Figure S32), whose performance and properties were analyzed by DMA, tensile testing, and contact angle, and oil sorption measurements (Figure 4h,i; Figures S33 and S34). No distinct variations were observed between the original and recycled coated **PET** fabrics, demonstrating the effectiveness of the chemical recycling method in the reuse of our textile sorbents.

## 3 Conclusions

In conclusion, we have presented a new type of textile sorbents by encapsulating commercially available fabrics, including PET, nylon, cotton, and polyimide, with dynamic imine networks, which are synthesized through a one‐pot polycondensation of terephthalaldehyde, isophorone diisocyanate, and a trifunctional amine cross‐linker. Following encapsulation, the textile composites exhibit improved thermal, thermomechanical, and mechanical properties as compared to both pristine and alkali‐etched fabrics. Specifically, these include an increased char ratio at 700 °C (25% *vs* 13%), a 2.3‐fold increase in *E*’ at 40 °C (77 *vs* 34 MPa), and an 8.6‐fold improvement in stress at break (12.9 *vs* 1.5 MPa) after the etched **PET** fabric was socked in 1 wt.% of **CPUI‐0.1** solution. The coating also imparts superhydrophobicity and antifouling resistance. A representative 1 wt.% **CPUI‐0.1**‐coated **PET** fabric displays a water contact angle of 136° and resists fouling from dyed and contaminated aqueous solutions, in stark contrast to neat **PET** fabrics that were readily wetted or stained. Importantly, the encapsulation with **CPUI‐0.1** endows these fabrics with excellent oil removal capability, increasing OSC by 40%–1520% depending on the oil and fabric types, coating concentration, and temperature. The most significant improvement was observed for nylon fabrics, where the OSC toward DCM increases from 0.22 g g^−1^ to 3.67 g g^−1^ after coating, an over 16‐fold enhancement. Of the same importance is the chemical recyclability of the **CPUI** coatings enabled by the dynamic imine chemistry. It allows the textile sorbents to be selectively depolymerized by an amine cross‐linker, recovering both the fabric substrates and the depolymerized coating solutions. Subsequent repolymerization and re‐coating refurnish the composites, which retain their mechanical strength, hydrophobicity, and oil sorption performance. Overall, the coated fabrics reported here are poised for rapid deployment, and the “encapsulation with dynamic covalent networks” strategy offers a versatile and sustainable platform in designing high‐performance, next‐generation textile sorbents, both contributing to a circular materials economy.

## Conflict of Interest

The authors declare no conflict of interest.

## Supporting information




**Supporting File 1**: advs73431‐sup‐0001‐SuppMat.pdf.

**Supporting File 2**: advs73431‐sup‐0002‐Video S1.mp4.

**Supporting File 3**: advs73431‐sup‐0003‐Video S2.mp4.

**Supporting File 4**: advs73431‐sup‐0004‐Video S3.mp4.

**Supporting File 5**: advs73431‐sup‐0005‐Video S4.mp4.

**Supporting File 6**: advs73431‐sup‐0006‐Video S5.mp4.

**Supporting File 7**: advs73431‐sup‐0007‐Video S6.mp4.

**Supporting File 8**: advs73431‐sup‐0008‐Video S7.mp4.

## Data Availability

The data that support the findings of this study are available from the corresponding author upon reasonable request.
