# Supplementary material for: Encapsulating Textiles with Dynamic Covalent Networks for Sustainable and Efficient Oil Spill Cleanup
Source: Adv Sci (Weinh). 2025 Dec 19;13(13):e20228. doi: 10.1002/advs.202520228 (PMC12955866; doi:10.1002/advs.202520228)
Supplement: Supplementary file 1 — Supporting File 1: advs73431‐sup‐0001‐SuppMat.pdf. [file ADVS-13-e20228-s007.pdf]

Encapsulating Textiles with Dynamic Covalent Networks for Sustainable and Efficient Oil Spill Cleanup

Changyi You[1#], Ping Yu[1#*], Haiyue Wang[1], Qirui Huang[1], Wei Hong[1], Cai Liu[1], Qinchao Sun[4], Yan Wang[3], Youwei Ma[2*], Zuming Hu[3]

[1] P. Yu, C. You, H. Wang, Q. Huang, W. Hong, C. Liu

School of Environmental Chemical Engineering, Jiangsu Ocean University, Lianyungang 222005, China

E-mail: yup@jou.edu.cn

[2] Y. Ma

Institute of Materials, École Polytechnique Fédérale de Lausanne (EPFL), Lausanne 1015, Switzerland

E-mail: youwei.ma@epfl.ch

[3] Z. Hu, Y. Wang

State Key Laboratory for Modification of Advanced Fiber Materials, College of Materials Science and Engineering, Donghua University, Shanghai 201620, China

[4] Q. Sun

Shandong Hualun Advanced Materials Co., Ltd., Linyi, Shandong 276600, China

[#]These authors contributed equally: Changyi You, Ping Yu

1. Experimental Section

Materials: Polyester, nylon, polyimide, and cotton fabrics were obtained from silk art workshop factory. Terephthalaldehyde (TA, 98%), *N*,*N*-dimethylformamide (DMF, ≥99.9%), chloroform ($CHCl_3$, 98%), and isophorone diisocyanate (IPDI, 99%) were purchased from Aladdin Reagent Co., Ltd., China. Trimethylolpropane tris[poly (propylene glycol), amine terminated] ether (T-403) and *n*-hexane (n-Hex, 99%) were obtained from Shanghai Adamas Co., Ltd., China. Ethanol (EtOH, AR), Dichloromethane (DCM, AR), *N*, *N*-Dimethylacetamide (DMAc, AR), and Petroleum ether (PE, AR) were purchased Yong Da Chemical Reagent Co., Ltd, China. Methanol (MeOH, 99.8%) was purchased Sinopharm Chemical Reagent Co., Ltd. Sudan I was purchased Shanghai Teng Zhun Biochemical Technology Co., Ltd, China. All reactions were carried out under standard laboratory conditions, with no pretreatments taken to exclude atmospheric moisture unless otherwise noted. Gear oil, hydraulic oil, brake fluid, automatic transmission fluid, lubricant, differential oil, and silicone oil all were obtained from Lianyungang Car Dealership.

Preparation of CPUIs Films: A series of CPUIs films were synthesized by mixing three reactive monomers including TA, IPDI, and T-403. The molar ratios of the monomers used in the synthesis of CPUIs were shown in Figure 1(b). Taking the synthesis of CPUI-0.1 as the example. First, TA (6.6 mmol, 0.886 g) and the solvent DMF (24.2 g) were mixed and dissolved in a 50 mL beaker by magnetic stirring. Then, T-403 (4.8 mmol, 2.112 g) was slowly added to the solution, and the mixture was stirred for 10 min. Next, IPDI (0.6 mmol, 0.134 g) was quickly added to the solution and stirred for 2 min. The solution was subsequently poured into a Teflon mold, and the solvent was evaporated in a drying oven at 60 °C for 18 h. The film was cooled to room temperature and peeled off from the Teflon mold. The film was then cut into a dumbbell shape and subjected to heat treatment in a vacuum oven at 150 °C for 1 h to obtain CPUI-0.1 with a thickness of approximately 0.2 mm.

Preparation of Coated fabrics: 10 g of sodium hydroxide were added to 800 mL of deionized water until complete dissolution. The PET fabric was then immersed in the solution and stirred at 75 ℃. After 2 h, the polyester fabric was removed and rinsed eight times with warm deionized water. The rinsed fabric was placed in a drying oven at 80 °C to dry. Next,

different weight ratios of CPUI-0.1 films in chloroform were prepared. The specific concentration of CPUI-0.1 used in the preparation of coated fabrics is shown in Table S1. Once the CPUI-0.1 films were completely dissolved, the dried alkali-treated polyester fabric was immersed in the solution for 12 hrs. After soaking, the fabric was placed in a drying oven at 80 °C to dry, to obtain coated fabrics. The coating content of the fabrics was calculated as follows, with the results shown in Table S1.

$$D\% = \frac{m_{Film}}{m_{Film} + m_{TCM}} \times 100\%$$

Table S1. Coating content of the coated PET fabrics.

**Samples**	**CPUI-0.1 concentration in $CHCl_3$ (wt%)**	**Coating content on the fabric (wt%)**
**Coated PET fabric**	0	0
**0.5 wt% coated PET fabric**	0.5	1.52
**1 wt% coated PET fabric**	1	6.03
**3 wt% coated PET fabric**	3	11
**5 wt% coated PET fabric**	5	26.3
**1 wt% coated nylon fabric**	1	8.8
**1 wt% coated cotton fabric**	1	6.1
**1 wt% coated polyimide fabric**	1	6.7

Characterizations: Fourier transform infrared spectroscopy (FTIR) was performed using a VERTEX 80 FTIR equipped with an attenuated total reflection (ATR) attachment. The scan range was from 600 to 4000 cm^{-1}, with 32 scans at a spectral resolution of 4.0 cm^{-1}. The FTIR spectra of IPDI, TA were performed using Thermo Fisher Scientific NicoletiS20. Temperature-variable FTIR of CPUI-1 using Nicolet iS50R FTIR were also recorded in the temperature ranging from 30 to 150 °C with a heating rate of 5 °C min^{-1}.

Atomic Force Microscopy (AFM) (Bruker, Dimension Icon) was used to obtain the height and phase images of film surface with a model of tapping.

Thermogravimetric analysis (TGA) was performed using a TGA 5500 (TA USA) instrument under a nitrogen (N_2) atmosphere. Each sample (5~10 mg) was heated from 30 to 800 °C at a rate of 20 °C min^{-1}.

Small Angle X-ray Scattering (SAXS) measurements were carried out using Xenocs Xeuss 3.0, with the sample-to-detector distance of 150 mm. The measurements were performed under vacuum, and at room temperature. The exposure time of the samples to the X-ray source was set as 1 h. Stretched and unstretched films were measured under identical conditions.

Differential scanning calorimetry (DSC) was conducted using on DSC 214 Polyma instrument with N_2 flow ranging from −30 to 150 °C, with a constant heating and cooling rate of 10 °C min^{-1}. The value of the glass transition temperature (T_g) was determined from the endothermic transition observed in the first heating curve.

Dynamic mechanical analysis (DMA) was performed using METTLER TOLEDO DMA1. The test was conducted with an oscillating frequency of 1 Hz. The samples were heated from −30 to 100 °C at a ramp rate of 5 °C min^{-1} to measure the storage modulus and loss factor (tan δ) as a function of temperature. Stress relaxation experiments were carried out on a Netzsch DMA 242E, using a tension film mode with a fixed frequency of 1 Hz and a preload force of 0.01 N. The samples were subjected to relaxation at a constant strain of 2% at temperatures of 50, 60, 70, and 80 °C for 20 min, respectively.

CPUIs were cut into small pieces and placed between two steel plates, each covered with a layer of polyethylene terephthalate (PET) film. The CPUIs films were then reprocessed through hot pressing at 80 °C and 10 MPa for 10 min.

Chemical recycling involves depolymerizing the CPUI-0.1 (0.373 g) in the presence of T-403 (1.478 g) at 60 ℃ using T-403 for 1 h, followed by adding specific amounts of TA (0.620 g) and IPDI (0.094 g). The mixture was first dried at 60 ℃ for 18 hrs and then at 150 °C under vacuum for 1 h to reproduce the recycled CPUI-0.1 film.

The mechanical measurements were conducted using a tensile tester (UTM4304-G Universal Tester with a 200 N transducer) at a temperature of 25 °C. Unless otherwise specified, all test specimens were dumbbell-shaped, with an effective length of 12 mm, width of 2 mm, and a measured thickness of approximately 0.2 mm. The tensile test was performed at a

constant speed of 2 mm min^{-1}, and at least five specimens were tested for each sample. The toughness of the samples was determined by calculating the area under the stress-strain curve, representing the energy absorbed during deformation. The cyclic tensile test was performed 10 times using INSTRON 5967, with the film stretched at a speed of 2 mm min^{-1} to a 10 % strain before unloading in 20 °C.

Coated PET fabric and etched PET fabrics were immersed in 3.5 wt% NaCl, DMF, NMP, EtOH and MeOH solutions for 24 hrs. Both dried and undried samples were subjected to tensile testing at 2 mm/min, and their tensile behaviors were observed.

To investigate the self-healing properties of CPUIs, optical microscopy (CPV-601C) was used to observe the material at different temperatures. First, a scratch was made on the surface of CPUI-0.5 film, which was then subjected to a heat treatment at 150 °C. The films were placed on glass slides and positioned on a heating stage. Finally, the scratch was monitored under an optical microscope as the temperature was increased to 70 °C and kept for 10 min.

The surface and cross-section of coated PET fabric, etched PET fabric and recycled coated PET fabric were observed using the German ZEISS Sigma 300 scanning electron microscope (SEM). All samples were gold-coated using the Quorum SC7620 sputtering coating instrument for 45 s. The distribution of oxygen elements on the surface of coated PET fabric was obtained using Energy dispersive spectroscopy (EDS) mapping analysis.

The hydrophobicity of the films was evaluated by measuring the water contact angle (WCA) using a contact angle analyzer (DSA100).

Oil Absorption Test: First, weigh a 1 cm × 1 cm sample of fabric to obtain mass m_1. Immerse the sample in different types of oil for 2 minutes, then take the sample out the oil, and let them rest for 10 seconds. Measure the weight of absorbed sample to obtain mass m_2. The oil absorption is calculated using the following formula,

$$w_i = \frac{m_2 - m_1}{m_1}$$

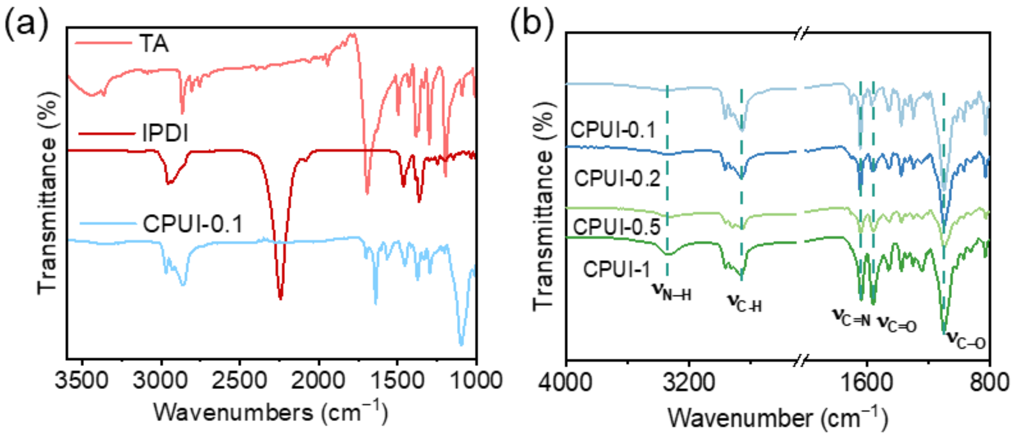

Figure S1. FTIR spectra of (a) IPDI, TA, and CPUI-0.1, and (b) the CPUI series.

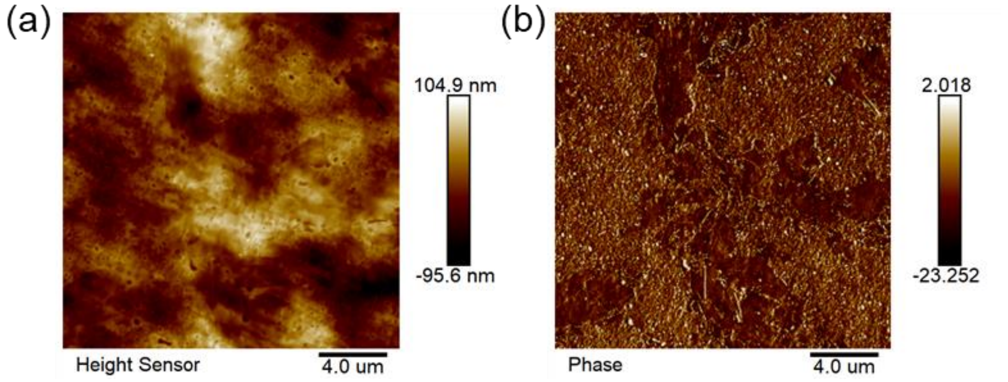

Figure S2. AFM height (a) and phase (b) images of the surface of CPUI-0.1.

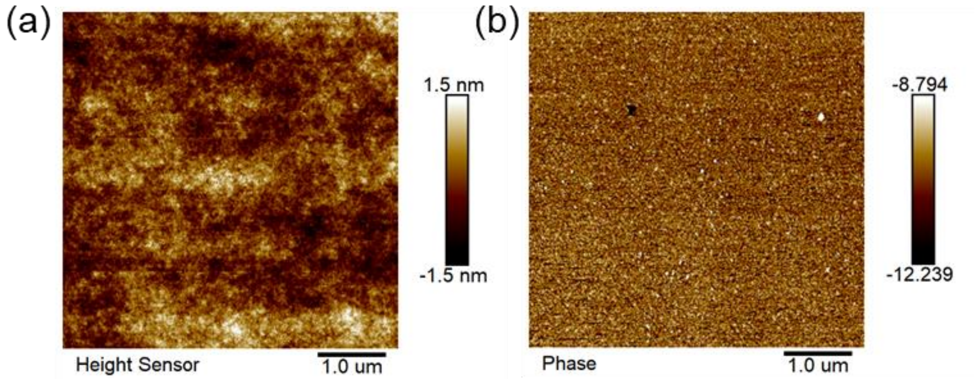

Figure S3. AFM height (a) and phase (b) images of the surface of CPUI-1.

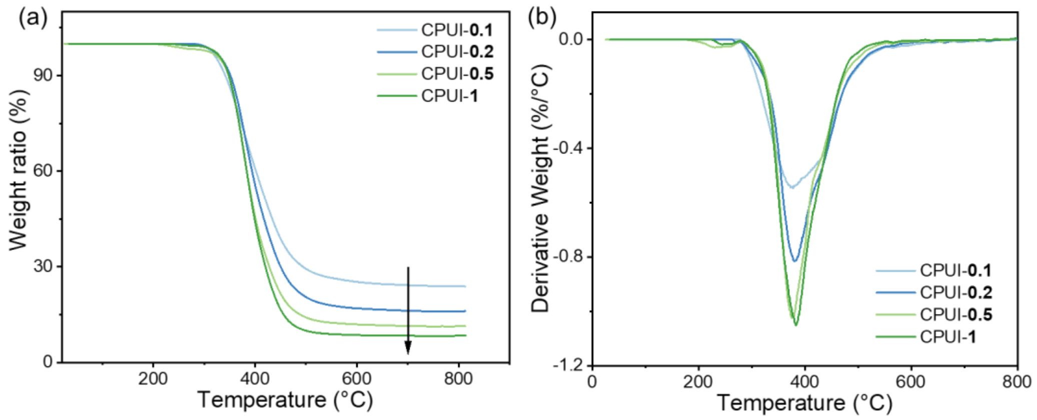

Figure S4. **(**a) TGA and (b) derivative thermogravimetry curves of the CPUI series.

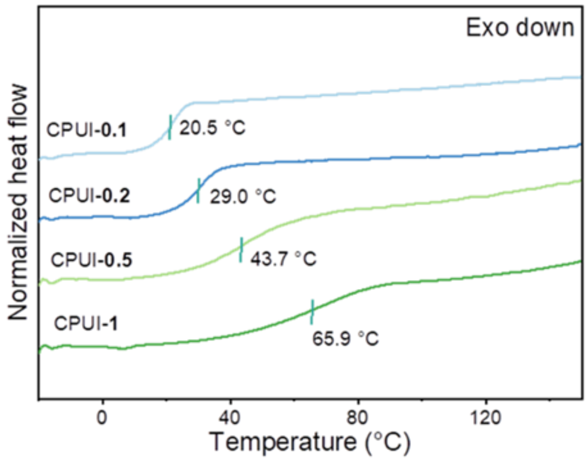

Figure S5. DSC traces of the second heating runs for the CPUI series.

(a)

Name	TA (mmol)	IPDI (mmol)	T-403 (mmol)
**Polyimine**	7.2	0	4.8
**Polyurea**	0	7.2	4.8

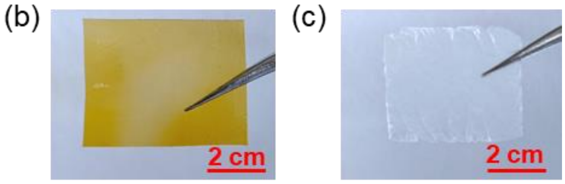

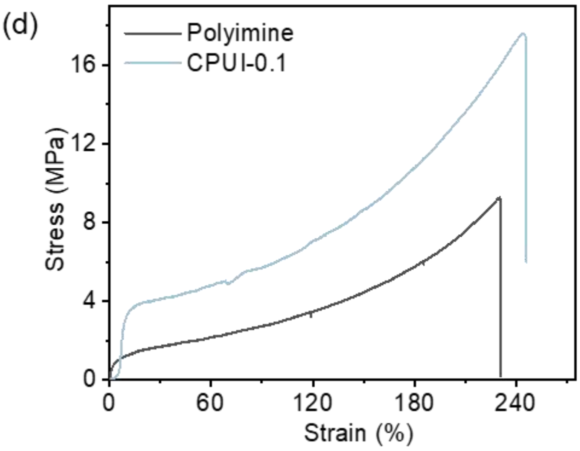

Figure S6. (a) Recipes used in the synthesis of polyimine and polyurea films. Photographs of a polyimine (b) and a polyurea (c) film. (d) Stress–strain curves of polyimine and CPUI-0.1 films.

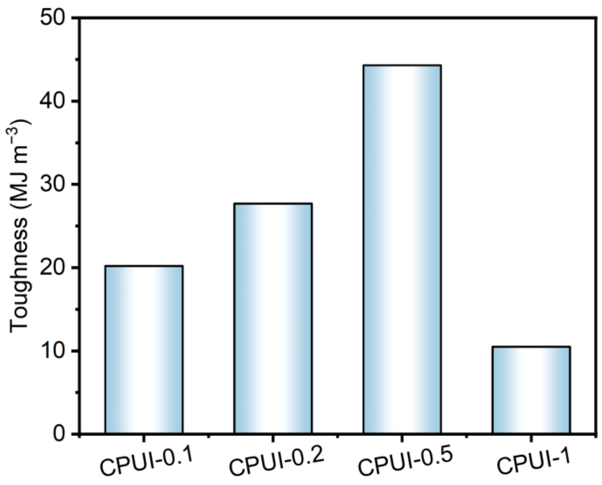

Figure S7. Toughness of CPUI-x films.

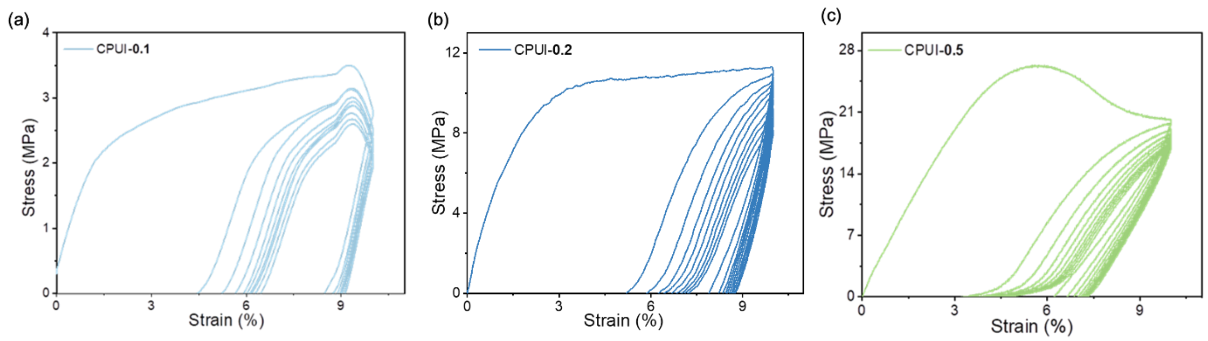

Figure S8. Cyclic tensile curves of (a) CPUI-0.1, (b) CPUI-0.2, and (c) CPUI-0.5 at a maximum loading strain of 10% for 10 consecutive cycles.

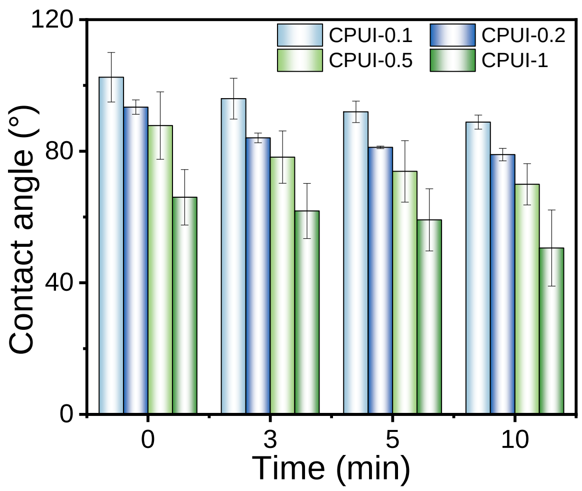

Figure S9. Histogram of the water contact angles of **CPUI-x** as a function of measuring time.

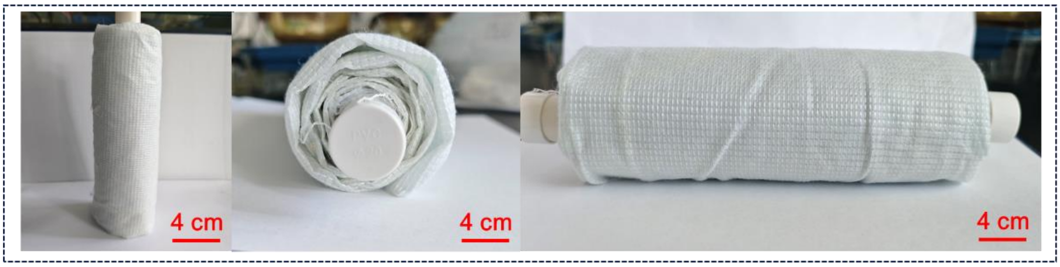

Figure S10. Photographs showing a roll of coated PET fabric, observed from different angles.

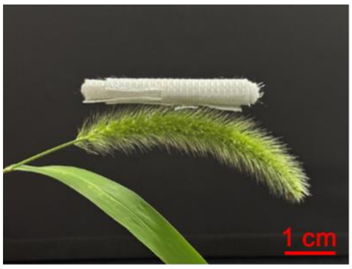

Figure S11. Photograph showing a roll of coated PET fabric with a weight of 0.44 g standing above a setaria viridis.

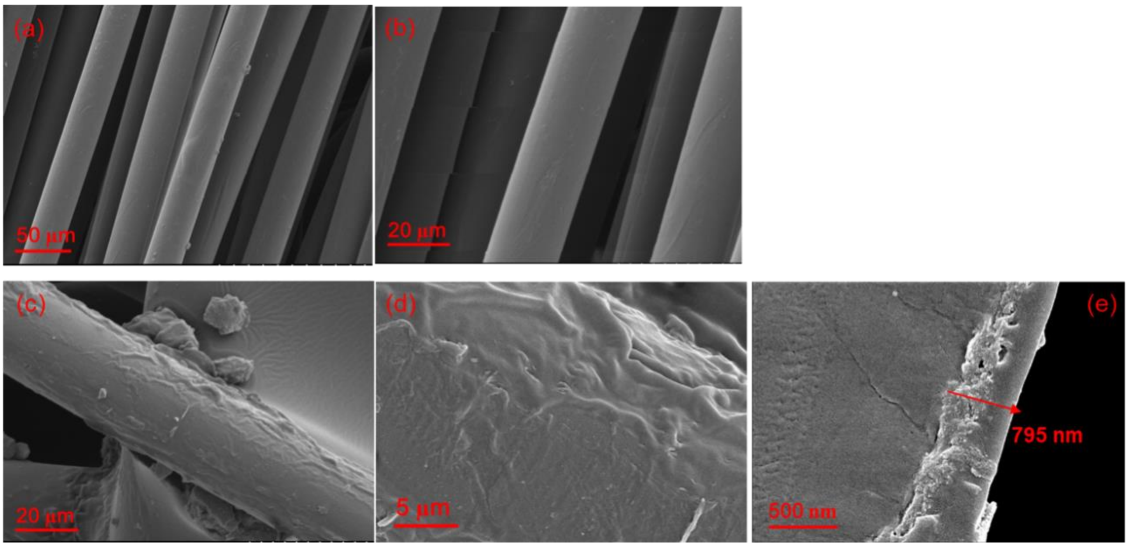

Figure S12. SEM images of the etched (a–b) and coated (c–d) PET fabrics at different magnifications. (e) SEM image of the cross-section of a coated PET fiber.

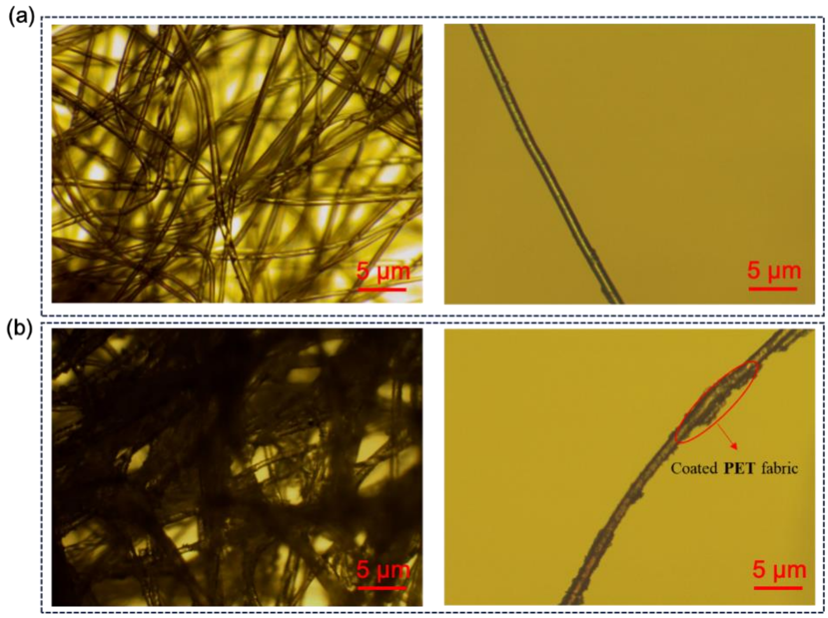

Figure S13. POM images of the etched (a) and coated (b) PET fabrics (left) and the corresponding single fiber (right).

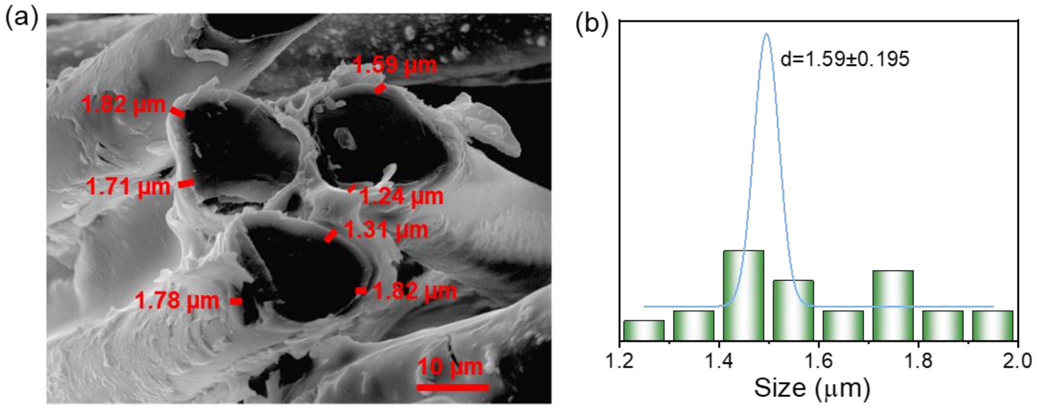

Figure S14. (a) SEM image of the cross-section of the **CPUI-0.1**-coated **PET** fibers. (b) Size distribution of the coating thickness.

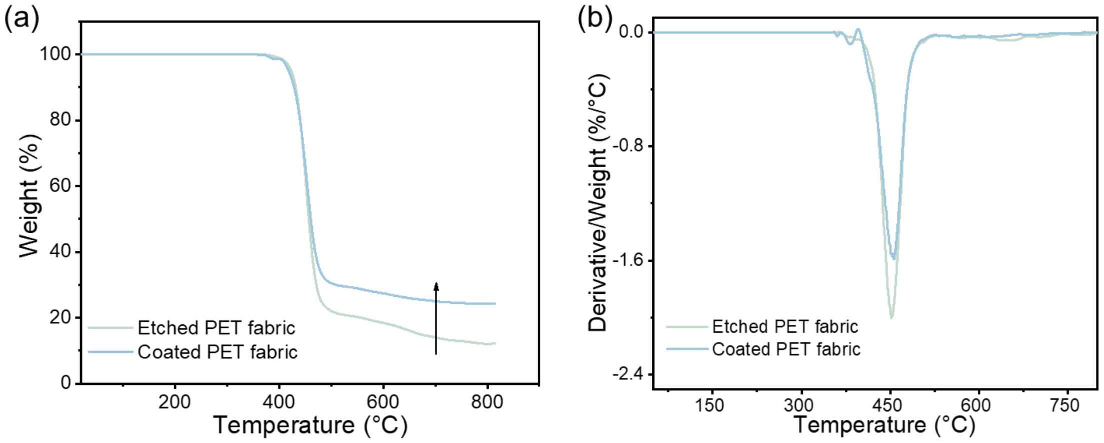

Figure S15. (a) TGA and (b) derivative thermogravimetry curves of the etched and CPUI-0.1-coated PET fabrics.

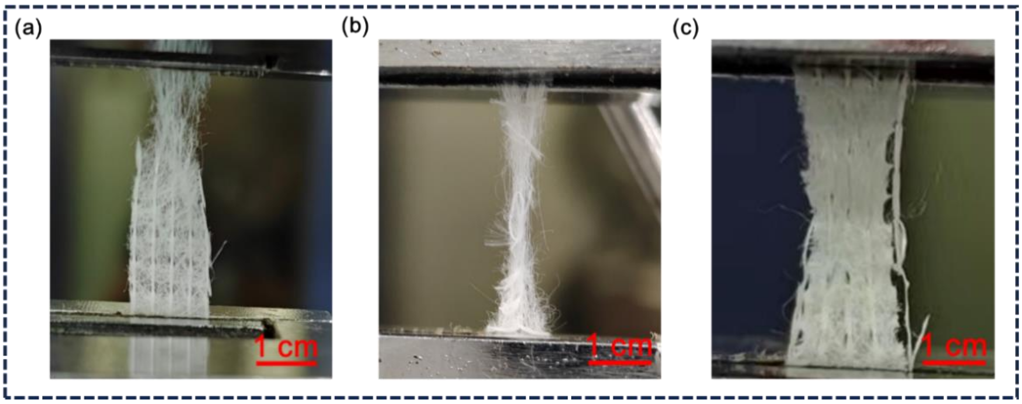

Figure S16. Photographs showing the stretching of the original (a), etched (b), and coated (c) PET fabrics during tensile testing measurements.

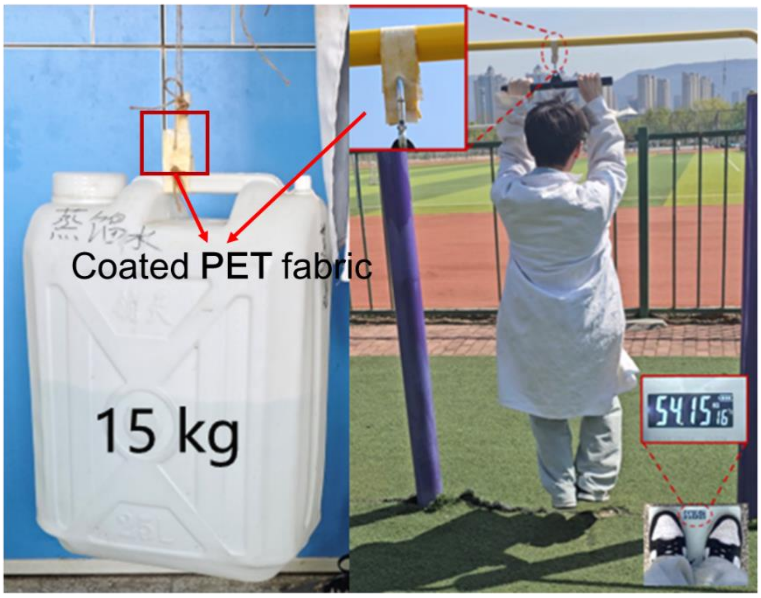

Figure S17. Photographs showing the coated fabric of a size of 9.6 cm ×2.4 cm × 0.15 cm to hold a 15 kg water bottle (left) and a 54 kg person (right).

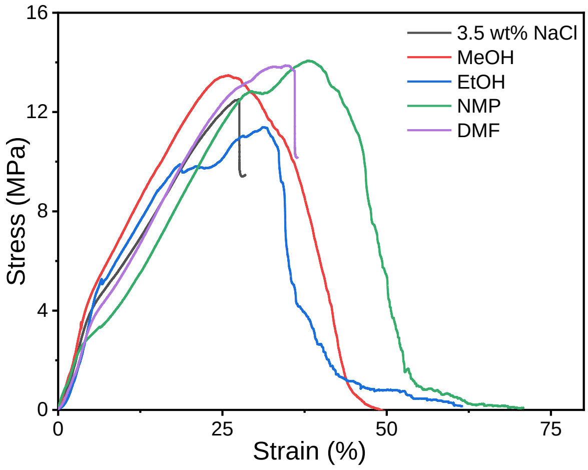

Figure S18. Stress–strain curve of the coated PET fabric immersed in various solvents and brine (3.5 wt% NaCl) for 24 h followed by drying at 70 °C for 2 h.

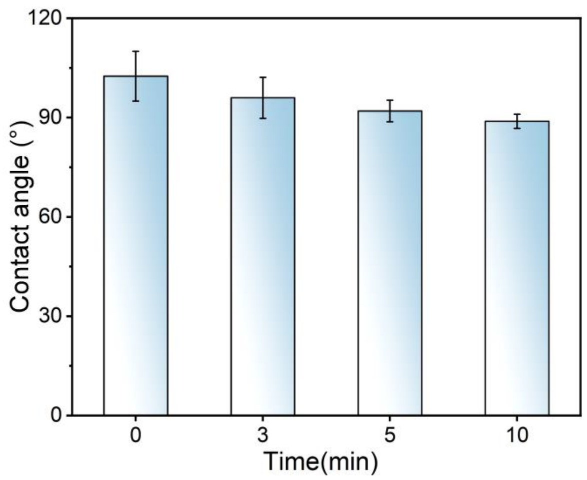

Figure S19. Histogram of the contact angles of CPUI-0.1 as a function of measuring time.

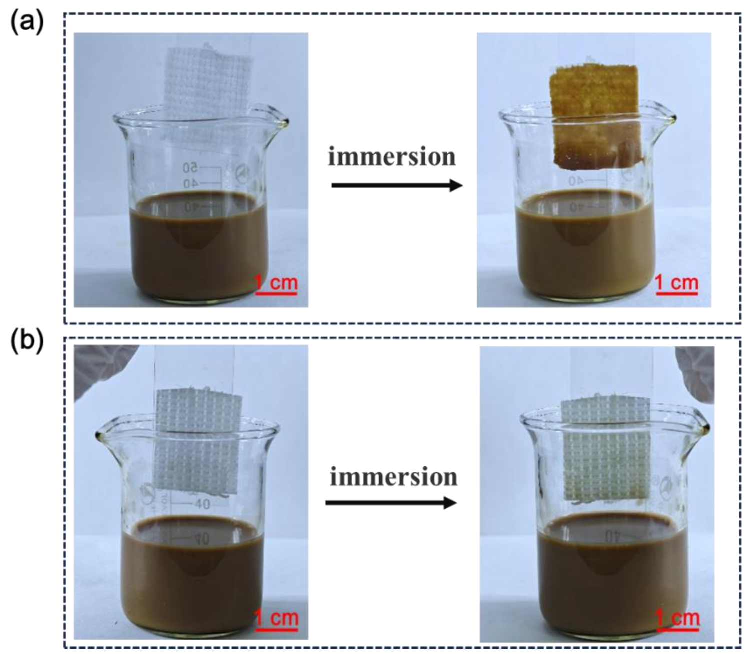

Figure S20. Photographs showing the original (a) and coated (b) PET fabrics before (left) and after (right) the immersion in coffee drink.

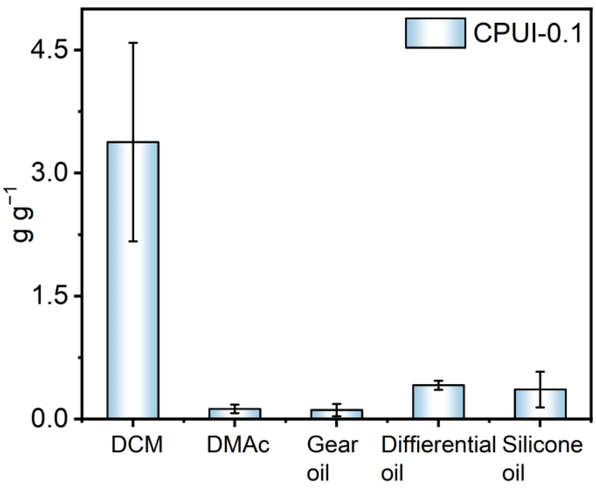

Figure S21. Histogram of the OSC of a CPUI-0.1 film toward organic solvents and industrial oils (as indicated).

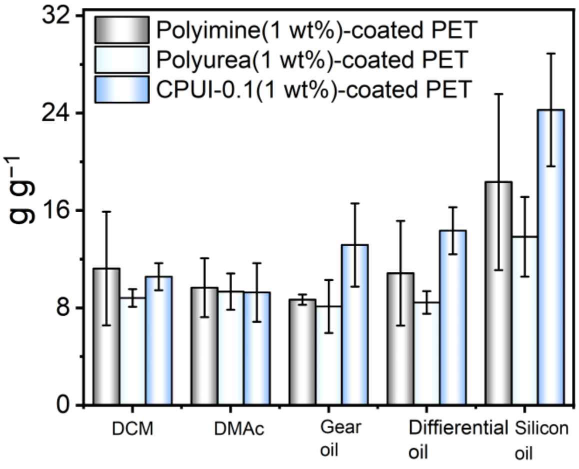

Figure S22. Histogram of the OSC of the neat polyimine-, polyurea-, and CPUI-0.1 (1wt%)-coated PET fabrics toward organic solvents and industrial oils (as indicated).

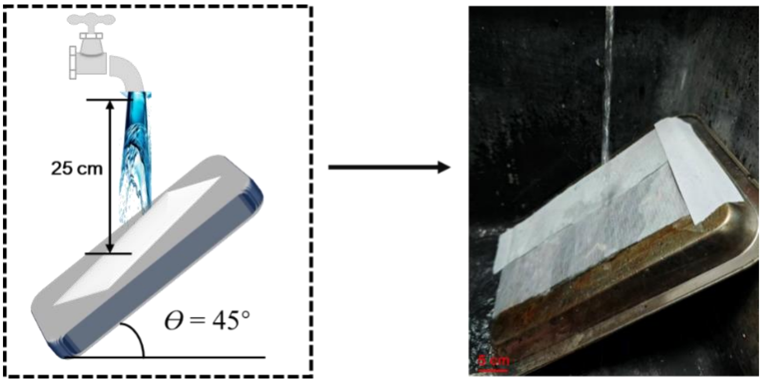

Figure S23. Simulated waving setup by pouring water onto the coated fabric from a height of 25 cm for 6 h.

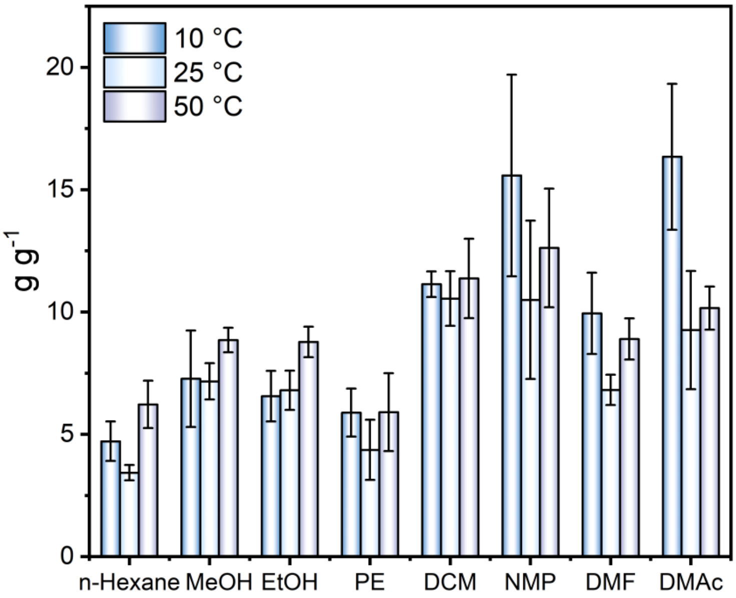

Figure S24. Histogram of the OSC of the CPUI-0.1 (1 wt%)-coated PET fabric measured at different temperatures.

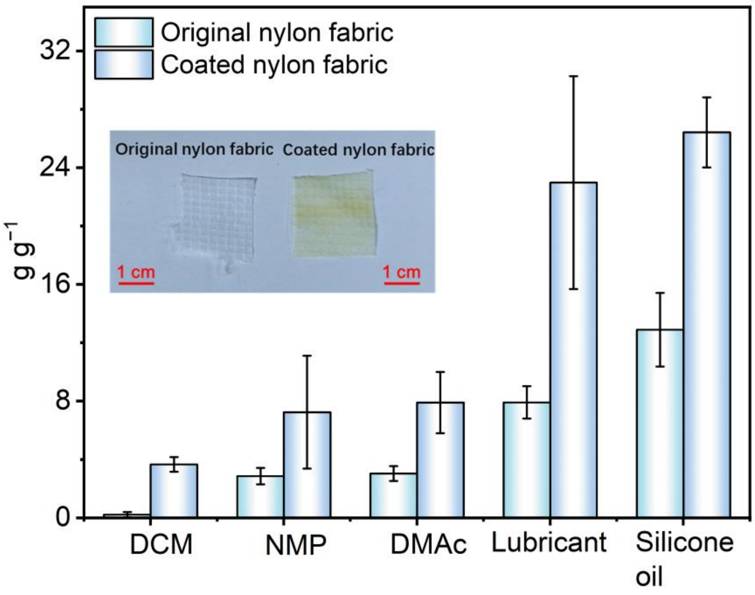

Figure S25. Histogram of the OSC of the original and CPUI-0.1 (1 wt%)-coated nylon fabrics.

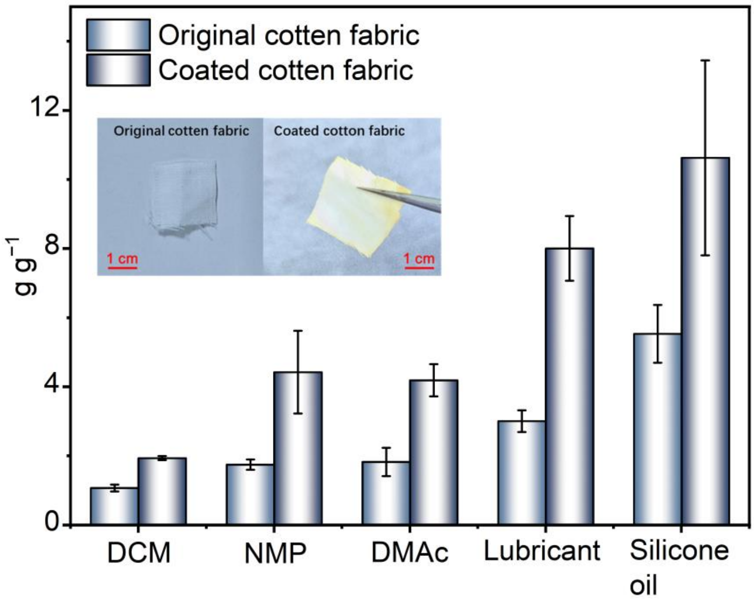

Figure S26. Histogram of the OSC of the original and CPUI-0.1 (1 wt%)-coated cotton fabrics.

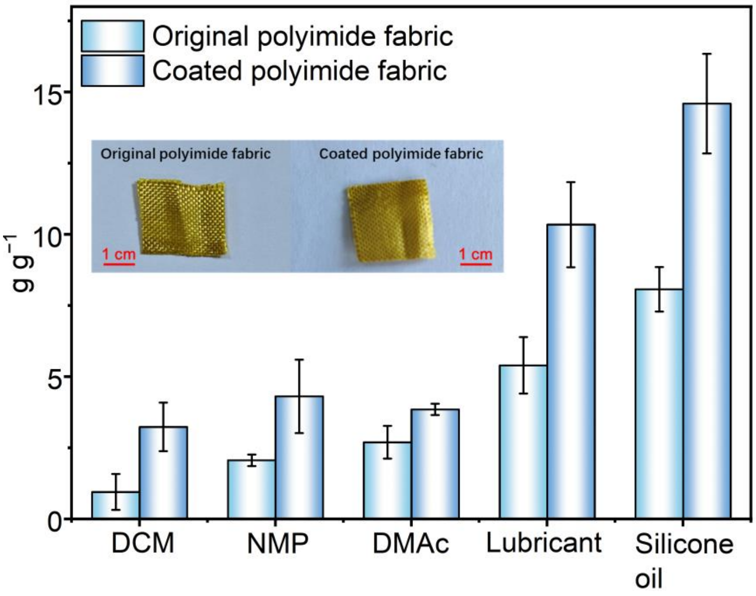

Figure S27. Histogram of the OSC of the original and CPUI-0.1 (1 wt%)-coated polyimide fabrics.

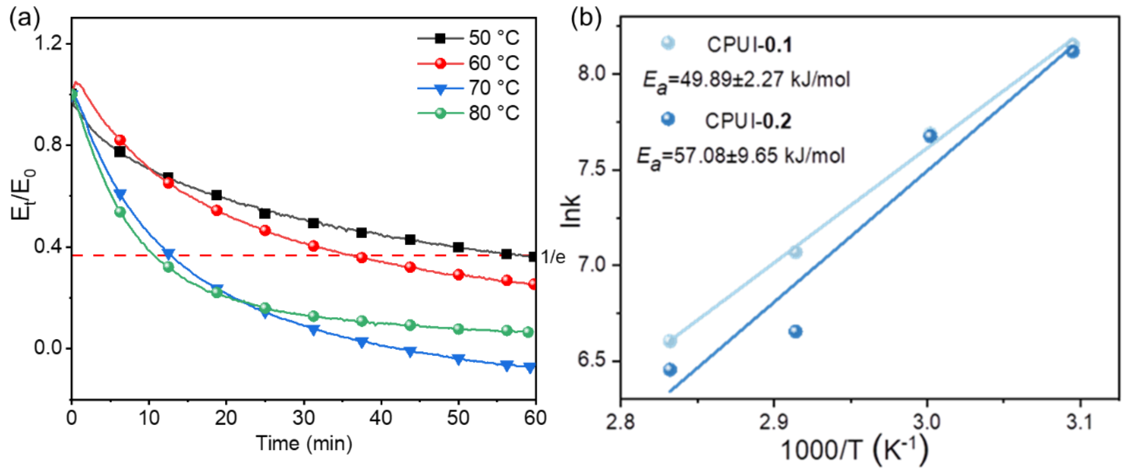

Figure S28. (a) Stress relaxation curves of CPUI-0.2 as a function of time. (b) Arrhenius plots for the stress–relaxation processes of CPUI-0.1 and CPUI-0.2.

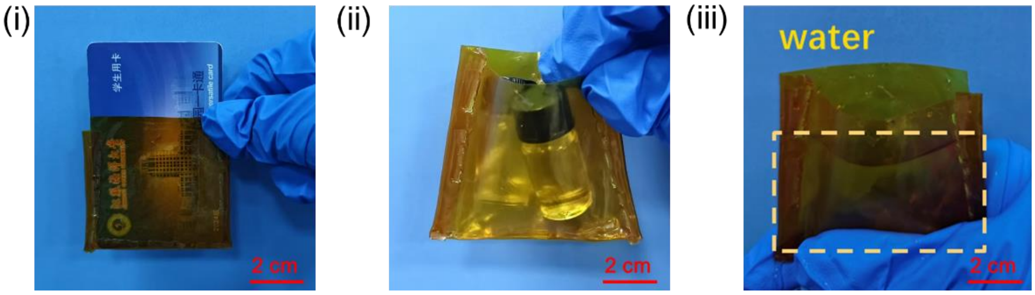

Figure S29. Photographs showing a plastic bag prepared by welding CPUI-0.1 films to hold a student card (a), a small vial (b), and water (c).

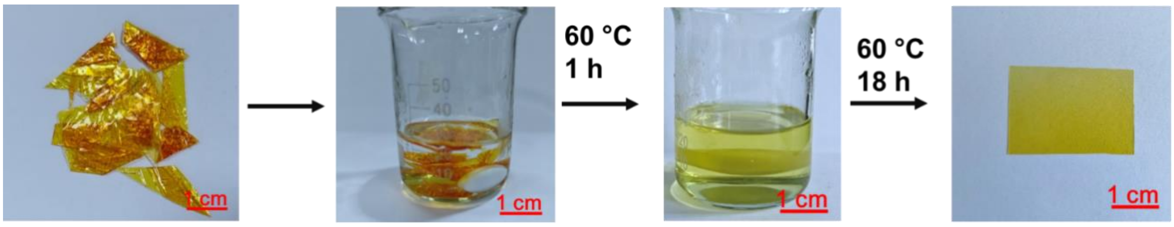

Figure S30. Photographs showing small pieces of CPUI-0.1 first immersed in T-403-containing DMF solution to form a clear solution upon heating at 60 °C for 1 h, followed by the addition of IPDI and TA, and subsequent heating at 60 °C for 18 h to reproduce a CPUI-0.1 film.

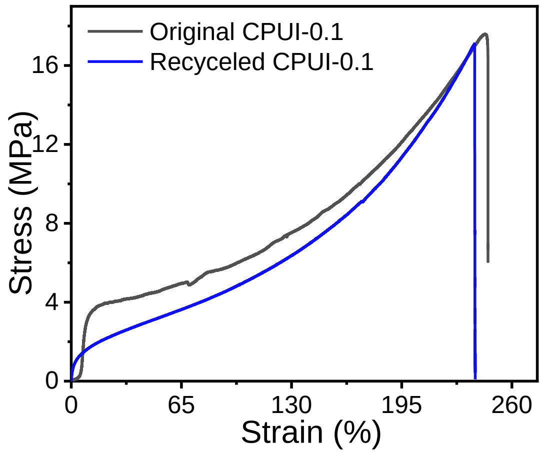

Figure S31. Stress–strain curves of the original and chemically recycled CPUI-0.1 films.

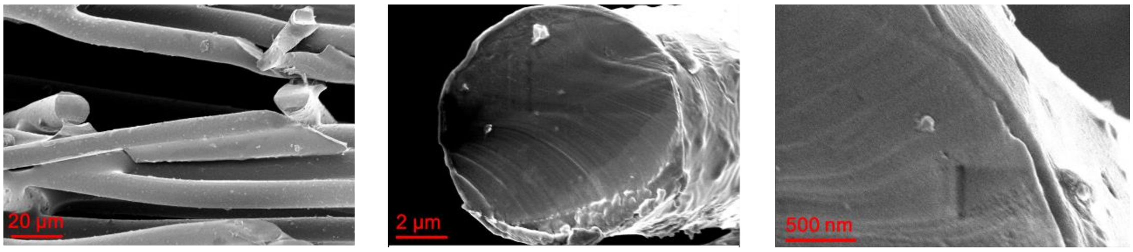

Figure S32. SEM images of the surface and cross-section (as indicated) of the recycled PET fabrics.

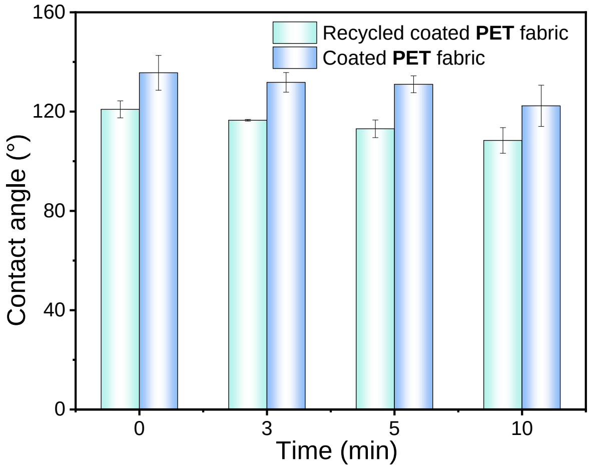

Figure S33. Comparison of water contact angles between the original and recycled coated PET fabrics.

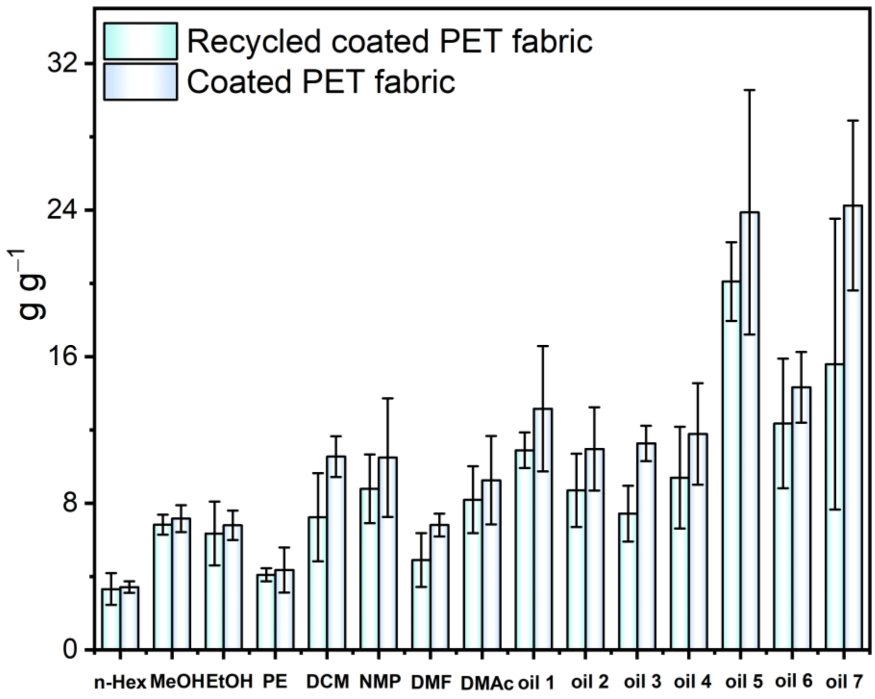

Figure S34. Comparison of OSC between the original and recycled coated PET fabrics. Oils 1–7 corresponds to gear oil, hydraulic oil, brake fluid, automatic transmission fluid, lubricant, differential oil, and silicone oil in sequence.

Table S2. Comparison on oil adsorption between this work and other recently reported works.[1-15]

**Material**	**Recyclability**	**Oil adsorption capacity (g/g)**	**Ref**
**Coated PET fabrics**	**Yes**	**~28.47 (Silicone) ~23.88(lubricating oil)**	**This work**
**POSS-CT**	No	~1.16-1.49 (Hexane)	*Int. J. Biol. Macromol.* **253**, 126748 (2023).
**HC-CEFF@C3**	No	~14.59 (DCM) ~16.32 (Silicone)	*Adv. Fiber Mater.* **6**, 1446–1455 (2024).
**PDMS/ZIF-8**	No	~24.5 (DCM)	*J. Environ. Chem. Eng.* **9**, 106329 (2021).

**Sa/HAP**	No	~12.13 (Pump oil)	*Chemosphere* **348**, 140651 (2024).
**Nanoporous PLAmicrofibers**	No	~26.78 (Pump oil)	*Sep. Purif. Technol.* **282**, 120156 (2022).
**PLA foam**	No	~24.5 (Silicone)	*ACS Appl. Mater. Interfaces* **11**, 14362–14367 (2019).
**PLA foam**	Yes	5-10 (Hexane)	*ACS Sustain. Chem. Eng*. **6**, 13834–13843 (2018).
**PLA**	No	~19.8 (pump oil)	*Chin. Chem. Lett.* **31**, 365-368 (2020).
**PLA/PBS$_2$foam**	No	~7.9 (Silicone)	*Sep. Purif. Technol.* **257**,117949 (2021).
**PP/mn PTFE**	No	~4.6 (Hexane)	*ACS Appl. Mater. Interfaces*, **11**, 7479–7487 (2019).
**PP nonwoven**	No	~21.99 (CCl_4)	*Polymer* **193**, 122356 (2020).
**ODTMS-HNTs**	No	~12.5 (CCl_4)	*Prog. Org. Coat.* **148**,105839 (2020).
**M-CT$_{15}$Z**	No	~21.79 (CH_3Cl_3)	*Carbohydr. Polym.* 369, 124283 (2025).
**SDPX**	No	~20 (Silicone)<br>~17 (Diesel)	*Adv. Funct. Mater.* **34**, 2313808 (2024).
**CAHB**	No	~8.852 (C_2H_5OH)	*J. Clean. Prod.* **524**, 146481 (2025).

Supplementary References

1. Lin, T-C. *et al.* Hydrophobic cotton fabric with 3-mercaptopropyltriethoxysilane/polyhedral oligomeric silsesquioxane/1-octadecanethiol modification for oil/water separation. *Int. J. Biol. Macromol.* **253** ,126748 (2023).
2. Yang, X. *et al.* Bioinspired design of textile-based absorbers: Photothermal and electrothermal synergistic conversion for efficient clean-up of heavy oil. *Adv. Fiber Mater.* **6**, 1446–1455 (2024).
3. Wang, J. *et al.* Recycled carbon fiber nonwoven functionalized with fluorine-free superhydrophobic PDMS/ZIF-8 coating for efficient oil-water separation. *J. Environ. Chem. Eng*. **9,** 106329 (2021).

4. Shafiq, F. *et al.* Stearic acid-modified hollow hydroxyapatite particles with enhanced hydrophobicity for oil adsorption from oil spills. *Chemosphere* **348**, 140651 (2024).
5. Zhang, L. *et al.* Development of highly oil-absorbent polylactic-acid microfibers with a nanoporous structure via simple one-step centrifugal spinning. *Sep. Purif. Technol.* **282**, 120156 (2022).
6. Wang, X. *et al.* Facile fabrication of superhydrophobic and eco-friendly poly(lactic acid) foam for oil–water separation via skin peeling. *ACS Appl. Mater. Interfaces* **11**, 14362–14367 (2019).
7. Wang, Y. *et al.* Recyclable oil-absorption foams via secondary phase separation. *ACS Sustain. Chem. Eng.* **6**, 13834–13843 (2018).
8. Wang, X. *et al.* Simple fabrication of superhydrophobic PLA with honeycomb-like structures for high-efficiency oil-water separation. *Chin. Chem. Lett.* **31**, 365–368 (2020).
9. Li, B. *et al.* Biodegradable PLA/PBS open-cell foam fabricated by supercritical CO_2 foaming for selective oil-adsorption. *Sep. Purif. Technol.* **257**,117949 (2021).
10. Peng, Y. *et al.* Tunable nonlinear acoustic reporters using micro- and nanosized air bubbles with porous polymeric hard shells. *ACS Appl. Mater. Interfaces* **11**, 7479–7487 (2019).
11. Zhang, H. *et al.* Groove-shaped polypropylene/polyester micro/nanofibrous nonwoven with enhanced oil wetting capability for high oil/water separation. *Polymer* **193**, 122356 (2020).
12. Song, Q. *et al.* Halloysite nanotubes functionalized cotton fabric for oil/water separation. *Prog. Org. Coat.* **148**, 105839 (2020).
13. Huang, W. *et al.* Tannic acid-assisted construction of ZIF-8/cellulose nanofiber composite aerogels for efficient oil adsorption and oil/water separation. *Carbohydr. Polym*. **369**, 124283 (2025).
14. Kimi, J. *et al.* Directional, silanized plant-based sponge for oil collection. *Adv. Funct. Mater.* **34**, 2313808 (2024).
15. Liang, D, et al. Construction of biochar-zeolite composite for enhanced VOCs adsorption: Interfacial reinforcement and steam-driven synergistic regulation of pore formation and dealumination. *J. Clean. Prod.* **524**, 146481 (2025).
